# Horizontal Plasmid Transfer among Klebsiella pneumoniae Isolates Is the Key Factor for Dissemination of Extended-Spectrum β-Lactamases among Children in Tanzania

**DOI:** 10.1128/mSphere.00428-20

**Published:** 2020-07-15

**Authors:** Torunn Pedersen, Marit Gjerde Tellevik, Øyvind Kommedal, Paul Christoffer Lindemann, Sabrina John Moyo, Jessin Janice, Bjørn Blomberg, Ørjan Samuelsen, Nina Langeland

**Affiliations:** a Norwegian National Advisory Unit on Detection of Antimicrobial Resistance, Department of Microbiology and Infection Control, University Hospital of North Norway, Tromsø, Norway; b Norwegian National Advisory Unit on Tropical Infectious Diseases, Department of Medicine, Haukeland University Hospital, Bergen, Norway; c Department of Microbiology, Haukeland University Hospital, Bergen, Norway; d Department of Clinical Science, University of Bergen, Bergen, Norway; e Department of Microbiology and Immunology, Muhimbili University of Health and Allied Sciences, Dar es Salaam, Tanzania; f Department of Medical Biology, Faculty of Health Sciences, UiT—The Arctic University of Norway, Tromsø, Norway; g Department of Pharmacy, Faculty of Health Sciences, UiT—The Arctic University of Norway, Tromsø, Norway; h Department of Medicine, Haraldsplass Deaconess Hospital, Bergen, Norway; JMI Laboratories

**Keywords:** CTX-M-15, HGT, IncFIIK5 plasmid, *Klebsiella pneumoniae*, whole-genome sequencing

## Abstract

Increased knowledge about the role of horizontal gene transfer is key to improve our understanding of the spread of antimicrobial resistance (AMR) in human populations. We therefore studied the dissemination of the *bla*_CTX-M-15_ extended-spectrum-β-lactamase (ESBL) gene in Klebsiella pneumoniae isolates obtained from stool samples from hospitalized children and healthy controls below 2 years of age in Dar es Salaam, Tanzania, from August 2010 to July 2011. We performed Illumina whole-genome sequencing (WGS) to characterize resistance genes, multilocus sequence type (MLST), plasmid incompatibility group (Inc), and plasmid MLST of 128 isolates of K. pneumoniae with *bla*_CTX-M-15_ recovered from both healthy and hospitalized children. We assessed the phylogenetic relationship using single nucleotide polymorphism (SNP)-based analysis and resolved the sequences of five reference plasmids by Oxford Nanopore technology to investigate plasmid dissemination. The WGS analyses revealed the presence of a *bla*_CTX-M-15_-positive IncFII_K5_/IncR plasmid with a highly conserved backbone in 70% (90/128) of the isolates. This plasmid, harboring genes encoding resistance to most β-lactams, aminoglycosides, trimethoprim-sulfamethoxazole, and chloramphenicol, was present in phylogenetically very diverse K. pneumoniae strains (48 different MLSTs) carried by both hospitalized and healthy children. Our data strongly suggest widespread horizontal transfer of this ESBL-carrying plasmid both in hospitals and in the general population.

**IMPORTANCE** Horizontal spread of plasmids carrying multiple resistance genes is considered an important mechanism behind the global health problem caused by multidrug-resistant bacteria. Nevertheless, knowledge about spread of plasmids in a community is limited. Our detailed molecular analyses of K. pneumoniae isolated from hospitalized and healthy children in Tanzania disclosed an epidemic spread of a resistance plasmid. In this study population, we revealed horizontal plasmid transfer among K. pneumoniae as the key factor for dissemination of ESBLs. Traditional outbreak investigation and surveillance focus on the spread of bacterial clones, and short-read sequencing can result in erroneous plasmid composition. Our approach using long-read sequencing reveals horizontal gene transfer of antimicrobial resistance, and therefore has a potential impact on outbreak investigations and approaches to limit spread of AMR.

## Abstract (precis)

Horizontal spread of plasmids carrying multiple resistance genes is considered an important mechanism behind the global health problem caused by multidrug-resistant bacteria. Nevertheless, knowledge about spread of plasmids in a community is limited. Our detailed molecular analyses of K. pneumoniae isolated from hospitalized and healthy children in Tanzania disclosed an epidemic spread of a resistance plasmid. In this study population, we revealed horizontal plasmid transfer among K. pneumoniae as the key factor for dissemination of ESBLs. Traditional outbreak investigation and surveillance focus on the spread of bacterial clones, and short-read sequencing can result in erroneous plasmid composition. Our approach using long-read sequencing reveals horizontal gene transfer of antimicrobial resistance, and therefore has a potential impact on outbreak investigations and approaches to limit spread of AMR.

## INTRODUCTION

The World Health Organization has declared infections caused by multidrug-resistant (MDR) bacteria an emerging global health problem of major public concern, as they undermine treatment for both common and life-threatening infectious diseases (1). Highly successful MDR bacterial clones disseminate worldwide (2). However, antibiotic resistance determinants may also spread horizontally by the transfer of mobile genetic elements, such as plasmids carrying multiple resistance genes (3). Although this phenomenon is well documented *in vitro*, we do not know how much horizontal gene transfer contributes to real-life spread of antimicrobial resistance (AMR) in a community. Horizontal gene transfer challenges traditional outbreak investigation and surveillance, which focuses on detecting the spread of bacterial clones based on their chromosomal DNA profiles.

Antimicrobial resistance spreads rapidly in resource-constrained settings, resulting in unacceptably high mortality from bacterial infections (4). We recently reported a high prevalence of fecal carriage of extended-spectrum-β-lactamase (ESBL)-producing MDR Klebsiella pneumoniae among both healthy and hospitalized Tanzanian children below 2 years of age (5). The most frequent ESBL type in this material was CTX-M-15, one of the most widespread and prevalent ESBL types worldwide (6). Among Tanzanian children, CTX-M-15 was a main cause of bloodstream infections and was associated with very high case-fatality rates (4, 7). To disentangle the underlying basis for this widespread dissemination of CTX-M-15, we employed long-read sequencing technology for circularization and tracking of an epidemic *bla*_CTX-M-15_-containing plasmid.

## RESULTS

### Nonclonal dissemination of CTX-M-15-positive K. pneumoniae.

Whole-genome sequencing (WGS) analyses of all ESBL-producing K. pneumoniae isolates revealed a diversity of resistance genes (see Table S1 in the supplemental material), confirming the MDR phenotype previously reported for the majority of strains (5). No carbapenemase-encoding genes were identified. However, all isolates were shown to carry the *bla*_CTX-M-15_ ESBL gene, which could suggest clonal dissemination.

10.1128/mSphere.00428-20.1 TABLE S1 Molecular characteristics of the K. pneumoniae strains. Download Table S1, DOCX file, 0.04 MB. Copyright © 2020 Pedersen et al. 2020 Pedersen et al. This content is distributed under the terms of the Creative Commons Attribution 4.0 International license.

Surprisingly, remarkably high diversity was observed at the bacterial genome level. Sixty-one different sequence types (STs) were identified, of which 32 were present only once (Table S1). No STs were encountered more than eight times, with ST336 (*n = *8), ST39 (*n = *7), ST14 (*n = *5), ST1552 (*n = *5), and ST3403 (*n = *5) being the most prevalent. Nine novel STs were identified.

**FIG 1:**
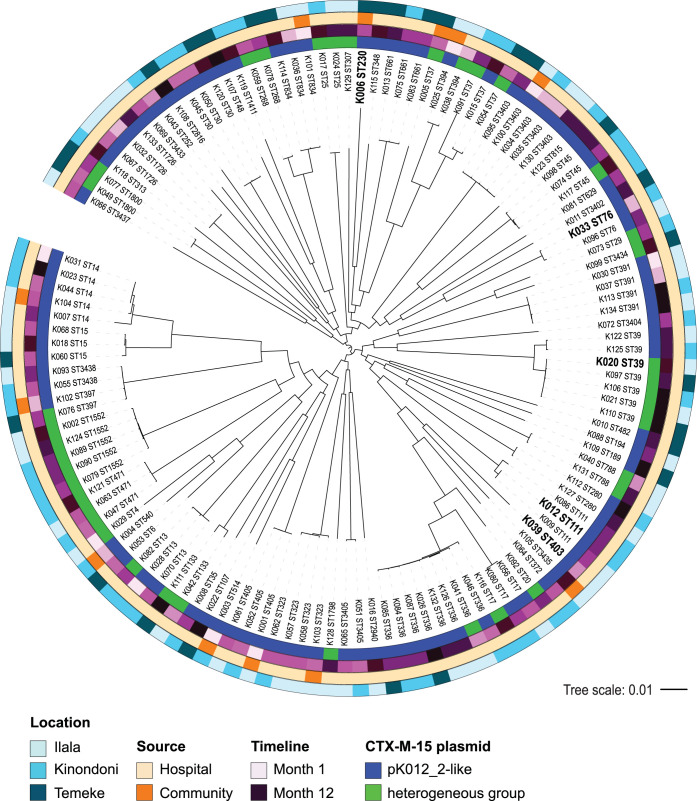
Phylogenetic tree of CTX-M-15-encoding K. pneumoniae (*n = *128) revealed by rapid core genome multialignment (https://github.com/marbl/parsnp). Metadata, including location of patient residence, source of isolation and time of sample collection within the 12-month study period (increased darkness of color), and group of plasmid, are visualized for each strain in the concentric rings (color codes as shown). Strains selected for long-read DNA sequencing are marked in bold. The tree is midpoint rooted with scale as shown.

**FIG 2:**
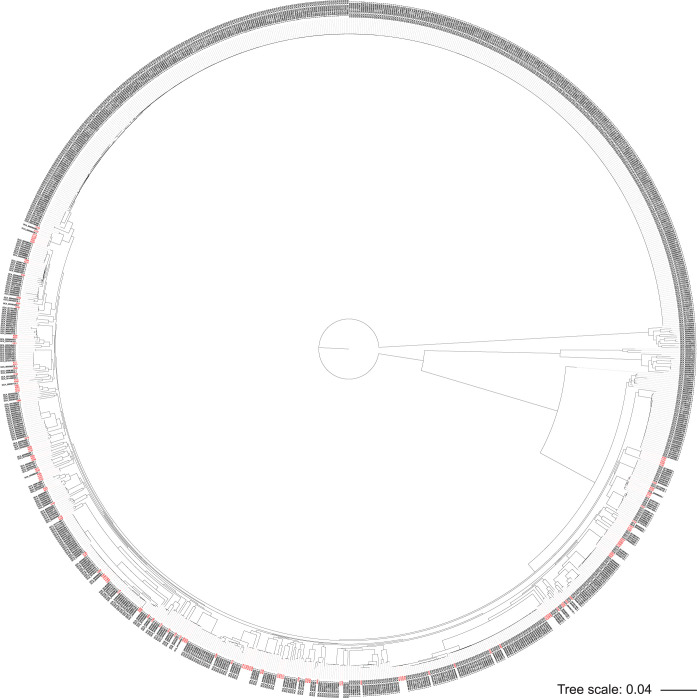
K. pneumoniae global phylogeny revealed by rapid core genome multialignment (https://github.com/marbl/parsnp). Assembly data set from this study (*n = *128; red) were analyzed together with data sets from the PATRIC database (*n = *970), for which GenBank assembly accession numbers are given for each of the PATRIC strains.

Core genome phylogenetic analyses confirmed the genomic diversity (Fig. 1). We observed no substantial clustering related to patient group (community or hospital), home district (Ilala, Kinondoni or Temeke), or time of sampling during the 1-year study period. Phylogenetic analyses, including genome assemblies from a global strain collection (*n = *1,098), revealed a wide distribution of the Tanzanian *bla*_CTX-M-15_-positive K. pneumoniae strains in the global tree (Fig. 2). Some were also part of clades constituting known global high-risk or outbreak clones, such as ST39, ST336, ST14/ST15, and ST405.

Thus, the dissemination of ESBL-producing K. pneumoniae among children in the Dar es Salaam region is linked to *bla*_CTX-M-15_ in a nonclonal population.

### *bla*_CTX-M-15_ is associated with IncFII/IncR plasmids.

The nonclonal nature of the K. pneumoniae population indicated that horizontal gene transfer played a major role in the dissemination of *bla*_CTX-M-15_. A large variety of different plasmid replicon types were observed within the collection (Table S1). However, all strains contained IncF replicons, of which 73% (94/128) belonged to the K5:A-:B IncFII subtype as determined by the plasmid multilocus sequence type (pMLST) and the FAB (FII:FIA:FIB) scheme. Moreover, all K5:A-:B-positive strains had an IncR replicon, which was also present in strains with other FAB types. In total, 10 different FAB types were detected. For three IncFIB(K)-positive strains a FAB type could not be determined.

10.1128/mSphere.00428-20.2 TABLE S2 Characteristics of the 128 CTX-M-15-positive K. pneumoniae carriers. Download Table S2, DOCX file, 0.01 MB. Copyright © 2020 Pedersen et al. 2020 Pedersen et al. This content is distributed under the terms of the Creative Commons Attribution 4.0 International license.

To identify the plasmids harboring *bla*_CTX-M-15_, we selected five strains (indicated in Fig. 1) from different STs for long-read sequencing. They represented both healthy and hospitalized children and all three home district locations. The antibiotic susceptibility profiles of the strains as determined by broth microdilution are shown in Table S3.

10.1128/mSphere.00428-20.3 TABLE S3 Susceptibility profiles for K. pneumoniae strains with completed genomes. Download Table S3, DOCX file, 0.02 MB. Copyright © 2020 Pedersen et al. 2020 Pedersen et al. This content is distributed under the terms of the Creative Commons Attribution 4.0 International license.

**FIG 3:**
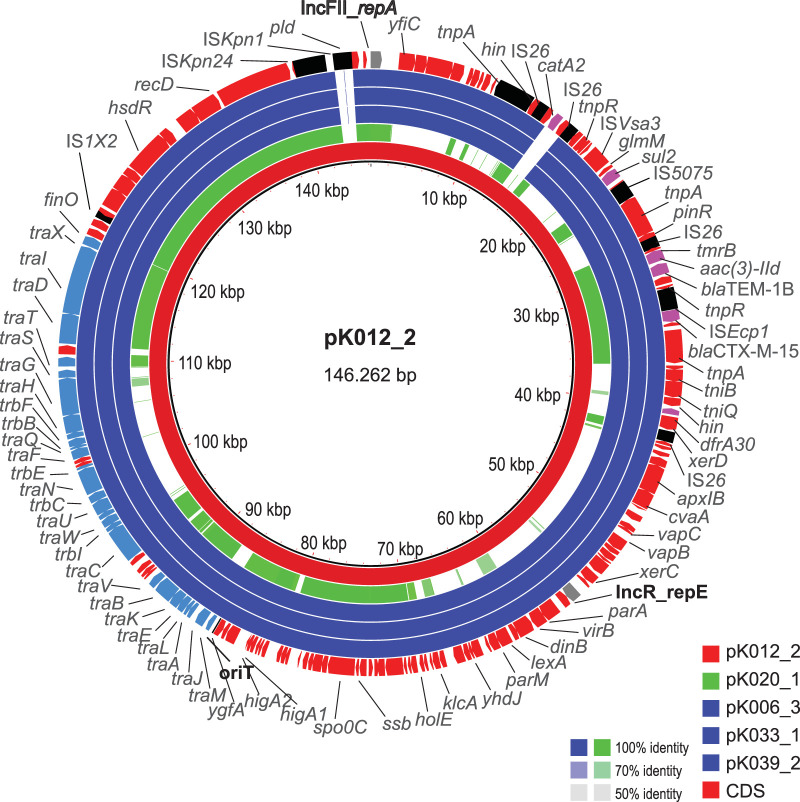
DNA alignment of *bla*_CTX-M-15_-containing plasmids. The concentric circles represent BLAST comparisons of plasmids pK020_1 (green) and pK006_3, pK033_1, and pK039_2 (dark blue) against the pK012_2 reference (red). The annotated coding DNA sequence (CDS) of the reference plasmid is shown in the outer circle, with the replication initiation encoding genes (gray), resistance-encoding genes (purple), IS elements (black), and F-conjugation module genes (light blue) highlighted. Color codes for DNA identity ranging from 100% to 50% as indicated. The map was constructed using the BLAST Ring Image Generator (http://brig.sourceforge.net/).

Circularization of CTX-M-15-encoding plasmids was obtained for four strains (K006, K012, K033, and K039). The corresponding plasmids (pK006_3 [143.593 bp], pK012_2 [146.262 bp], pK033_1 [150.094 bp], and pK039_2 [142.041 bp]) were of similar sizes and contained both the IncFII (K5:A-:B) and IncR replicons. They harbored a variety of resistance genes and insertion sequence (IS) elements as well as an F-like conjugation module (Fig. 3). The only identifiable differences between the four plasmids were the absence of IS*Kpn1* in three of them and variability in the *catA2* region delineated by IS*26*, which have an impact on their total size. An additional copy of IS*26* bordered the entire resistance module, in which *bla*_CTX-M-15_ was situated next to IS*Ecp1B* and in proximity to *bla*_TEM-1B_.

**FIG 4:**
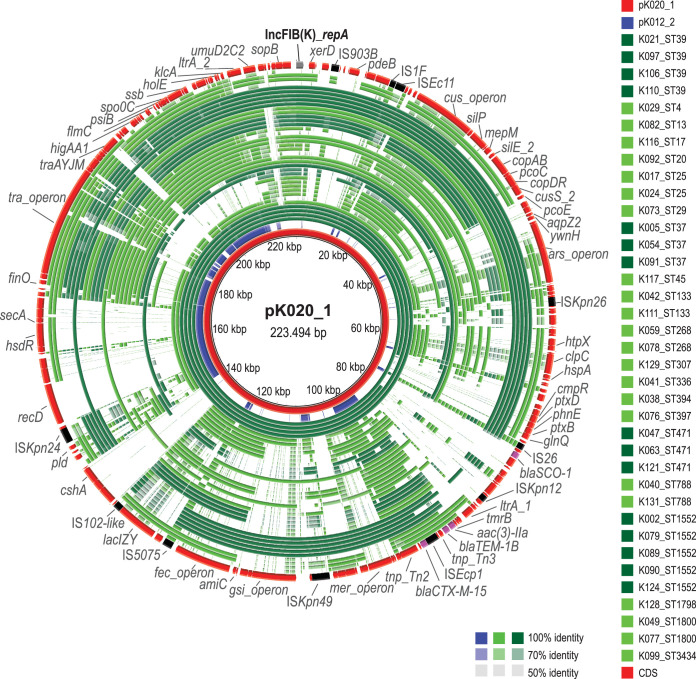
Tracking of CTX-M-15-encoding plasmid sequences by using pK020_1 as a reference. The green concentric circles (*n = *37) represent BLAST comparisons of the genome assemblies (strains listed) against the reference, starting with the innermost circle. Color codes for strains belonging to STs which include three or more strains, and for DNA identity ranging from 100% to 50% identity, are indicated. The red circles represent the reference, including annotated CDS in which replication initiation encoding genes (gray), resistance-encoding genes (purple), and IS elements (black) are highlighted.

For strain K020, we obtained a noncircular CTX-M-15-encoding plasmid sequence (pK020_1; 223.494 bp) of the IncFIB(K) replicon type (Fig. 4). Although sharing regions, including the MDR region containing *bla*_CTX-M-15_, sequence alignment showed that the overall gene synteny and genetic content differed from those of pK012_2.

Overall, the plasmid sequencing identified a conserved IncFII(K5:A-:B)/IncR *bla*_CTX-M-15_-containing plasmid in four of the strains. In the last strain, *bla*_CTX-M-15_ was carried by a completely different plasmid structure.

### Horizontal spread of a CTX-M-15-encoding IncFII/IncR plasmid.

**FIG 5:**
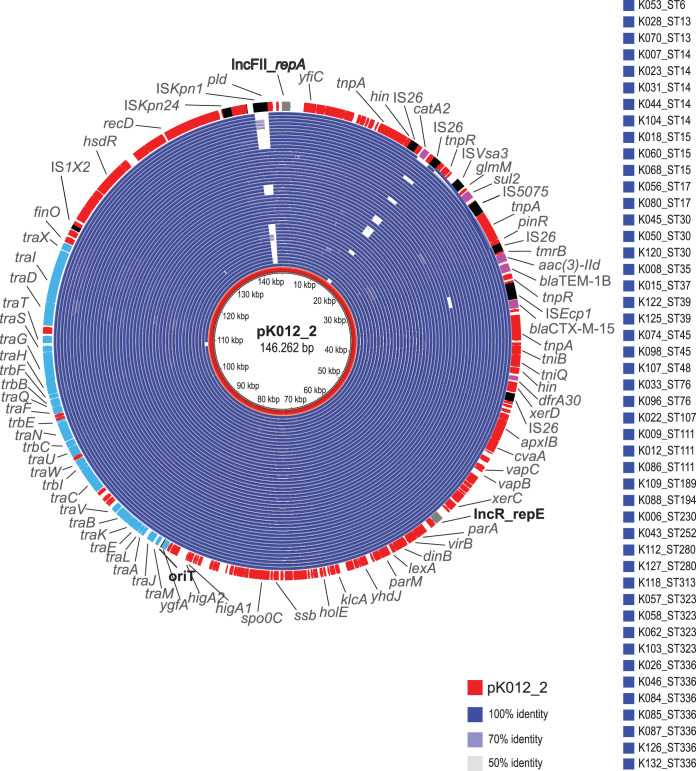

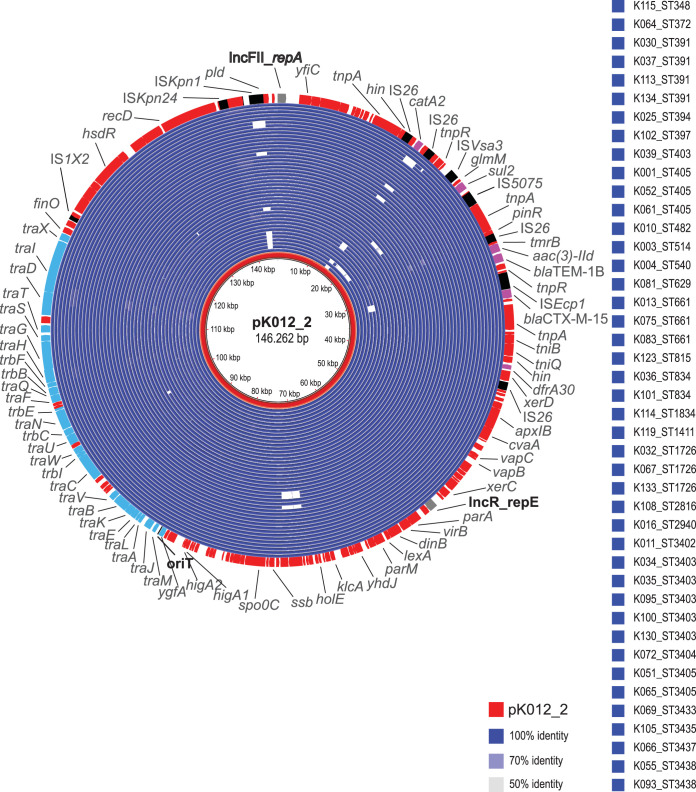
Tracking of CTX-M-15-encoding plasmid sequences by using pK012_2 as a reference. The blue concentric circles (*n = *90) represent BLAST comparisons of the genome assemblies against the reference plasmid, starting with the first of the listed strains as the innermost circle. Color codes for DNA identity ranging from 100% to 50% are indicated. The red circles represent the reference, including annotated CDS in which replication initiation-encoding genes (gray), resistance-encoding genes (purple), IS elements (black), and F-conjugation module genes (light blue) are highlighted.

To investigate the prevalence of the two identified types of plasmids, each of them was used as a reference in BLAST comparisons with all the genome assemblies (*n = *128). We identified DNA sequences highly similar to pK012_2 in 90 (70%) of them (Fig. 5). The presence of mobile genetic elements, IS*Kpn* and *catA2* delineated by IS*26*, represents the main variations detected. Intriguingly, the strains harboring the pK012_2-like plasmids belong to 48 different STs, which strongly implies horizontal spread.

**FIG 6:**
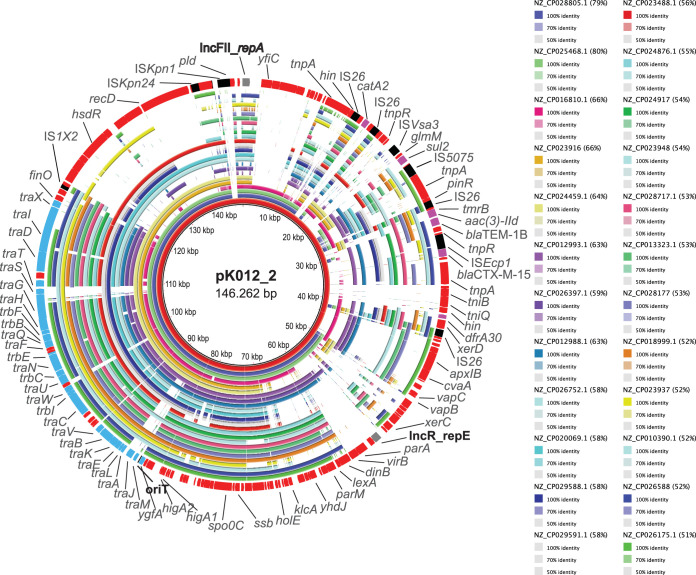
Comparison of pK012_2 with completed plasmids from the NCBI reference database. The concentric multicolored circles represent BLAST comparisons of selected plasmids having an overall coverage of >50% and DNA identity of >97% (*n = *24; color codes as indicated) correlated to pK012_2. Accession numbers and color codes for each of the plasmids are given. The red circles represent the pK012_2 reference plasmid, including annotated CDS in which replication initiation-encoding genes (gray), resistance-encoding genes (purple), IS elements (black), and F-conjugation module genes (light blue) are highlighted.

The origin and overall dissemination of pK012_2-like plasmids were assessed by a BLAST search in the NCBI database. As visualized in Fig. 6, including all BLAST hits with DNA coverage of >50%, no highly similar plasmids were found.

For the remaining 38 genome assemblies, BLAST comparison against pK020_1 revealed considerable variation in DNA coverage (Fig. 4), showing that *bla*_CTX-M-15_ is contained by a heterogeneous group of plasmids. For the strains belonging to the same ST as the pK020_1 reference plasmid (ST39), we tracked DNA sequences with high identity and coverage (four innermost rings). In other STs, variable parts of the reference plasmid were represented. The pattern of DNA coverage was most similar among phylogenetically related strains, implying clonal spread.

As shown in Fig. 1, several phylogenetic clusters (ST13, ST17, ST37, ST39, ST45, ST336, ST394, and ST397) included both pK012_2-containing strains and strains in which *bla*_CTX-M-15_ was carried by plasmids assigned to the heterogeneous group.

Taken together, our findings imply independent resistance acquisition by genetically unrelated strains concerted by local development and clonal dissemination. The main contributor to the spread of ESBL in this strain collection is one plasmid (pK012_2), which can be termed an outbreak plasmid.

## DISCUSSION

Considering the long inclusion period and recruitment of both healthy and hospitalized children from geographically distant clinics, the phylogenetically widely diverse CTX-M-15-positive K. pneumoniae strains were not surprising. However, it is remarkable that identical or nearly identical plasmids were widely dispersed among these diverse bacterial strains obtained from children during a whole year, regardless of which districts the children lived in and whether they were healthy or hospitalized. Our finding, due to the use of long-read sequencing, is explained by horizontal gene transfer of an “epidemic plasmid” over a relatively long period. This could have important implications for AMR surveillance and outbreak investigations since traditional genetic methods leave plasmid dissemination undetected, and short-read sequencing can result in erroneous conclusions regarding the actual plasmid composition and content (8).

Despite the age of the collected strains, our findings add new details to the understanding of how horizontal spread of successful plasmids can shape bacterial populations and affect surveillance of antibiotic resistance. We do not know how long the identified plasmid has circulated in the Tanzanian population and what the current situation is. The fact that it was far more common in hospitalized than in healthy children indicates that it spreads particularly well in hospital environments. The stool samples were collected within 24 h after admission for hospitalized children, and the *bla*_CTX-M-15_-containing plasmids were far more common among the very youngest children, i.e., those under 3 months of age. This indicates that hospital delivery may be a risk factor for carriage, although we had not collected sufficient information to confirm this hypothesis. Other risk factors could be prior antibiotic usage and other illnesses predisposing for hospitalizations. We do not know whether such horizontal gene transfer of resistance occurs in environments with less crowding, better sanitation, and better hospital infection control measures.

The dominant plasmid identified in the present investigation belongs to the IncFII_K_ group, found to be prevalent in K. pneumoniae but sparse in other *Enterobacterales* (9). The IncFIIK group of plasmids share common features, including an F-like conjugation module enabling horizontal transfer and the presence of additional replicons. Most often, IncFII_K_ plasmids are of narrow host range when carrying other IncF replicons (9).

The plasmid additionally carried IncR, which is frequently detected together with IncF on chimeric plasmids in *Enterobacterales* (10). Plasmids fusions between IncFII and IncR can be mediated during antibiotic pressure (11), and cointegration of different replicons is suggested as an adaptive mechanism for extending the host range and thereby plasmid survival and dissemination (10). *bla*_CTX-M-15_ has been associated with both IncR and IncFII_K_ plasmids as well as an IncR/IncFII_K_ multireplicon plasmid (11–15). The observed linkage of *bla*_CTX-M-15_ to IS*ECp1* is frequently detected and has proven to contribute to a high level of *bla*_CTX-M-15_ expression as well as mobilization (11, 16, 17). *bla*_CTX-M-15_ was embedded in a genetic region bracketed by IS*26*, which is known to reorganize plasmids by replicative transposition or by homologous recombination and also to mediate duplication of resistance-encoding genes (14, 18). The region covered all plasmid-borne resistance genes, including *bla*_TEM-1_, which is commonly associated with *bla*_CTX-M-15_ (19, 20), and contained additional IS elements, including IS*26*. The multiple layers of mobile units add complexity to the development of this *bla*_CTX-M-15_ plasmid and also provide further potential for mobilization and recombination.

Carrying a plasmid will normally impose a fitness cost to a bacterium. However, over time, the bacterium can accumulate compensatory mutations that alleviate this cost (21). An environment with high antibiotic pressure may enable bacterial clones to acquire AMR-encoding plasmids and favors the spread of low-cost plasmids carrying a well-adapted selection of AMR-encoding genes. This would create more dynamic and successful plasmid-bacterium associations that could gradually increase the relative contribution of plasmid-mediated horizontal gene transfer to the spread of AMR in a population. The wide dissemination of the plasmid across unrelated bacterial clones favors the concept of a well-adapted, low-cost plasmid over the concept of well-adapted carrier bacteria. This is in line with a recent study that concluded, based on mathematical modeling and simulation, that plasmid-located compensatory mutations are more effective than chromosomal mutations in supporting plasmid persistence and spread (22).

In conclusion, our findings reveal dissemination of CTX-M-15-encoding K. pneumoniae due to horizontal plasmid transfer rather than clonal dissemination and emphasize the limitations of outbreak investigations based on whole-genome phylogeny alone.

## MATERIALS AND METHODS

### Ethics statement.

The Muhimbili University of Health and Allied Sciences Institutional Review Board in Tanzania, the Regional Committee for Medical and Health Research Ethics (REK 2010/2564) in Norway, and the hospital authorities at the three study hospitals approved the study. Written informed consent was obtained from the parents or guardians on behalf of all the children enrolled in the study.

### Study population.

The present work was part of an unmatched case-control study assessing the causes of diarrhea among children, in which 1,287 participants below 2 years of age were recruited. The study population and data collection have previously been described (5, 23). The study was conducted between August 2010 and July 2011 in Dar es Salaam, the largest city in Tanzania, with a population of more than four million. Study participants were healthy community children attending child health clinics for immunization and growth monitoring with no history of diarrhea for 1 month prior to the study enrollment (*n *= 250), children hospitalized due to diarrhea (*n *= 250), and children admitted due to diseases other than diarrhea (*n = *103). Study hospitals were three major hospitals of Dar es Salaam: Muhimbili National Hospital, Amana Regional Hospital, and Temeke Regional Referral Hospital. For hospitalized children, the sample was collected within the first 24 h upon admission. Screening for the carriage of ESBL in stool was part of the original protocol. We screened half of the study population (*n *= 603; selected to be representative for the total study population) for fecal carriage of ESBL-producing *Enterobacterales* and reported a high fecal carriage of ESBL-producing and multidrug-resistant bacteria (5).

Characteristics of the study population are shown in Table S2. The majority of the carriers of CTX-M-15-positive K. pneumoniae were less than 1 year of age. ESBL carriage was found among both hospitalized and community children, but with a much higher carriage prevalence among hospitalized children. Recent antibiotic use was reported for a large proportion of participants. Risk factors for ESBL carriage have been described previously (5).

### Sample material and selection of isolates.

Stool specimens were collected directly after inclusion in the study and cultured within 6 h. Specimens were shipped on dry ice to Bergen, Norway, for further analysis (5). For the current study, we included all 128 CTX-M-15-positive K. pneumoniae isolates available from the initial study (5). One isolate was missing and hence not included. Antimicrobial susceptibility testing was performed using broth microdilution with premade plates (TREK Diagnostic Systems/Thermo Fisher Scientific, East Grinstead, UK).

### DNA sequencing.

Genomic DNA for Illumina sequencing was extracted from overnight bacterial colonies using the MagNA Pure 96 DNA and Viral NA Large Volume kit (Roche Diagnostics GmbH, Mannheim, Germany) according to the manufacturer’s instructions. Genomic libraries were prepared using the Nextera XT DNA library preparation kit (Illumina, San Diego, CA), and 150-bp paired-end sequencing was performed using the HiSeq 4000 system or the MiSeq system (Illumina). The obtained sequencing results are given for the individual samples in Table S4.

10.1128/mSphere.00428-20.4 TABLE S4 Whole-genome sequencing data and NCBI accession numbers for the K. pneumoniae strains. Download Table S4, DOCX file, 0.03 MB. Copyright © 2020 Pedersen et al. 2020 Pedersen et al. This content is distributed under the terms of the Creative Commons Attribution 4.0 International license.

For sequencing by MinION from Oxford Nanopore Technologies (ONT; Oxford, UK), genomic DNA was extracted by using the Genomic-tip 100/G kit (Qiagen, Hilden, Germany) and DNA fragments below 3 to 4 kb were removed by using AMPure XP beads (A63882; Beckman Coulter, Krefeld, Germany), both according to the manufacturers’ instructions. Libraries were prepared using a rapid barcoding kit (SQK-RBK001) and sequenced on R9.4 flow cells (FLO-MIN106), both supplied by ONT.

### DNA sequence analysis.

Illumina raw reads were assembled using SPAdes 3.9, and ONT long-read sequences were assembled together with Illumina raw reads using Unicycler (https://omictools.com/unicycler-tool) (24). ResFinder 3.0, MLST 1.8 or 2.0, PlasmidFinder 1.3 or 2.0, and pMLST 2.0, available from the Center for Genomic Epidemiology (https://cge.cbs.dtu.dk/services/), were applied to identify acquired resistance genes, multilocus sequence types (MLSTs), plasmid replicon types, and plasmid MLSTs (pMLST) (25–27). Prokka was applied for annotation of the completed genomes (28). By using Circlator v1.5.4, *dnaA* (chromosome) or *repA* (plasmids) genes were set at the first nucleotide positions (29). Plasmids were additionally curated by using ISfinder (https://isfinder.biotoul.fr). Assemblies deposited in GenBank were annotated using the PGAP pipeline provided by the NCBI (https://www.ncbi.nlm.nih.gov/genome/annotation_prok/).

The phylogenetic relationship was investigated by running the Illumina data sets (*n = *128) through Parsnp v.1.2 with “–c” flags enabled and random reference selection among the included samples (30). FigTree (http://tree.bio.ed.ac.uk/software/figtree/) was used to visualize and edit the tree. Metadata, including plasmid group, origin of isolate (community or hospital), timeline, and geographic origin (district; patient residence), were included as outer circles by the use of iTOL software (https://itol.embl.de/). For global phylogeny, selected K. pneumoniae (with fewer than 200 contigs and known MLST) genomes (*n = *970) downloaded from GenBank using metadata from the PATRIC database (https://www.patricbrc.org/) were included in the Parsnp analyses.

The housekeeping genes of unknown STs were sent for curation and assignment at the K. pneumoniae MLST database at the Pasteur Institute.

To search for the presence of specific plasmid sequences, selected plasmids were used as reference input to BLAST Ring Image Generator (BRIG) together with Illumina assemblies (31). Except for shading (false), default values were used for all BRIG parameters.

### Accession numbers.

The raw read sequences have been deposited in GenBank under BioProject no. PRJNA503964. Plasmid sequences have accession numbers CP034319.1 (pK006_3), RSEZ01000005.1 (pK012_2), SBIL01000003.1 (pK020_1), CP034322.1 (pK033_1), and CP034361.1 (pK039_2).
