# Supplementary material for: Horizontal Plasmid Transfer among Klebsiella pneumoniae Isolates Is the Key Factor for Dissemination of Extended-Spectrum β-Lactamases among Children in Tanzania
Source: mSphere. 2020 Jul 15;5(4):e00428-20. doi: 10.1128/mSphere.00428-20 (PMC7364214; doi:10.1128/mSphere.00428-20)
Supplement: TABLE S1 [file mSphere.00428-20-st001.docx]

**TABLE S1** Molecular characteristics of the *K. pneumoniae* strains

| **ID­-Study group** |  | **ST^[[1]](#footnote-1)^** | **Plasmid replicons** |  | **Genes encoding resistance to:** | | | |
| --- | --- | --- | --- | --- | --- | --- | --- | --- |
|  |  |  |  | **pMLST (IncF^[[2]](#footnote-2)^)** | **Beta-lactams** | **Aminoglycosides** | **Co-trimoxazole** | **Other agents** |
| K001-C^[[3]](#footnote-3)^ |  | 405 | IncFIB(K); IncFII(K); IncR; ColRNAI | K5:A-:B- | *bla*_TEM-1B_; *bla*_CTX-M-15_; *bla*_SHV-76_ | *aac(3)-IId* | *dfrA14*; *dfrA30*; *sul2* | *oqxA*; *oqxB*; *fosA*; *mph(A)*; *catA2* |
| K002-H^[[4]](#footnote-4)^ |  | 1552 | IncFIB(K); IncFII(K); IncR; ColRNAI | K1:A-:B- | *bla*_TEM-1B_; *bla*_CTX-M-15_; *bla*_SHV-62_-like | *aac(3)-IId* | *dfrA30*; *sul2* | *oqxA*; *oqxB*; *fosA*; *catA1* |
| K003-C |  | 514 | IncFIB(K); IncFII(K); IncR; ColRNAI | K5:A-:B- | *bla*_TEM-1B_; *bla*_CTX-M-15_; *bla*_SHV-63_ | *aac(3)-IId*; *aph(3’’)-Ib; aph(6)-Id* | *dfrA7*; *dfrA30*; *sul1*; *sul2* | *oqxA*; *oqxB*; *fosA*; *catA2; erm(C)* |
| K004-C |  | 540 | IncFIB(K); IncFII(K); IncR; ColRNAI | K5:A-:B- | *bla*_TEM-1B_; *bla*_CTX-M-15_ | *aac(3)-IId* | *dfrA30; sul2* | *oqxA*; *oqxB*; *fosA*; *catA2; erm(C)* |
| K005-C |  | 37 | IncFIB(K); IncFII(K); IncR; ColRNAI | F-:A13:B- | *bla*_TEM-1B_; *bla*_CTX-M-15_; *bla*_SHV-11_; *bla*_OXA-1_ | *aac(6')Ib-cr*; *aac(3)-IIa*; *aph(6)-Id* | *dfrA14*; *sul2* | *oqxA*; *oqxB*; *qnrB1*; *fosA*; *mph(A)*; *catA1*; *catA2*; *tet(D)* |
| K006-C |  | 230 | IncFIB(K); IncFII(K); IncR; ColRNAI | K5:A-:B- | *bla*_TEM-1B_; *bla*_CTX-M-15_; *bla*_SHV-27_ | *aac(3)-IId; aph(3’’)-Ib* | *dfrA30*; *sul2* | *oqxA*; *oqxB*; *fosA* |
| K007-H |  | 14 | IncFIB(K); IncFII(K); IncR; ColRNAI | K5:A-:B- | *bla*_TEM-1B_; *bla*_CTX-M-15_; *bla*_SHV-28_ | *aac(3)-IId* | *dfrA30*; *sul2* | *oqxA*; *oqxB*; *fosA*; *catA2; erm(C)* |
| K008-H |  | 35 | IncFIB(K); IncFII(K); IncR; ColRNAI | K5:A-:B- | *bla*_TEM-1B_; *bla*_CTX-M-15_; *bla*_SHV-33_-like | *aac(3)-IId*; *aph(3’’)-Ib*; *aph(6)-Id* | *dfrA22*; *dfrA30*; *sul1*; *sul2* | *oqxA*; *oqxB*; *fosA*; *catA2* |
| K009-H |  | 111 | IncFIB(K); IncFII(K); IncR; ColRNAI | K5:A-:B- | *bla*_TEM-1B_; *bla*_CTX-M-15_; *bla*_SHV-11_ | *aac(3)-IId* | *dfrA30*; *sul2* | *oqxA*; *oqxB*; *fosA*; *catA2* |
| K010-H |  | 482 | IncFIB(K); IncFII(K); IncR; ColRNAI | K5:A-:B- | *bla*_TEM-1B_; *bla*_CTX-M-15_; *bla*_SHV-27_ | *aac(3)-IId* | *dfrA30*; *sul2* | *oqxA*; *oqxB*; *fosA5*; *catA2* |
| K011-H |  | 3402 | IncFIB(K); IncFII(K); IncR; ColRNAI | K5:A-:B- | *bla*_TEM-1B_; *bla*_CTX-M-15_; *bla*_SHV-38_ | *aac(3)-IId* | *dfrA30*; *sul2* | *fosA* |
| K012-H |  | 111 | IncFIB(K); IncFII(K); IncR; ColRNAI | K5:A-:B- | *bla*_TEM-1B_; *bla*_CTX-M-15_; *bla*_SHV-11_ | *aac(3)-IId* | *dfrA30*; *sul2* | *oqxA*; *oqxB*; *fosA*; *catA2* |
| K013-H |  | 661 | IncFIB(K); IncFII(K); IncR; ColRNAI | K5:A-:B- | *bla*_TEM-1B_; *bla*_CTX-M-15_; *bla*_SHV-27_ | *aac(3)-IId* | *dfrA30*; *sul2* | *oqxA*; *oqxB*; *fosA*; *catA2* |
| K015-H |  | 37 | IncFIB(K); IncFII(K); IncR; ColRNAI | K5:A-:B- | *bla*_TEM-1B_; *bla*_CTX-M-15_; *bla*_SHV-11_ | *aac(3)-IId*; *aph(3‘’)-Ib*; *aph(6)-Id* | *dfrA30*; *sul2* | *oqxA*; *oqxB*; *fosA*; *catA2*; *erm(C)* |
| K016-H |  | 2940 | IncFIB(K); IncFII(K); IncR; ColRNAI | K5:A-:B- | *bla*_TEM-1B_; *bla*_CTX-M-15_; *bla*_SHV-27_ | *aac(3)-IId* | *dfrA30*; *sul2* | *oqxA*; *oqxB*; *fosA*; *catA2* |
| K017-H |  | 25 | IncFIB(K); IncFII(K); IncR; ColRNAI | nd^[[5]](#footnote-5)^ | *bla*_TEM-1B_; *bla*_CTX-M-15_; *bla*_SHV-11_ | *aac(3)-IIa*; *aph(3’’)-Ib*; *aph(6)-Id* | *dfrA25*; *sul1*; *sul2* | *oqxA*; *oqxB*; *qnrB2*; *fosA*; *catA2*; *tet(D)*; *erm(C)* |
| K018-H |  | 15 | IncFIB(K); IncFII(K); IncR; ColRNAI | K5:A-:B- | *bla*_TEM-1B_; *bla*_CTX-M-15_; *bla*_SHV-28_ | *aac(3)-IId*; *aadA1*; *aph(6)-Id* | *dfrA15; dfrA30; sul1; sul2* | *oqxA*; *oqxB*; *fosA*; *catA2*; *tet(A)* |
| K020-H |  | 39 | IncFIB(K); IncFII(K); IncR; ColRNAI | K2:A-:B- | *bla*_TEM-1B_; *bla*_CTX-M-15_; *bla*_SHV-12_; *bla*_SCO-1_ | *aac(3)-IIa; aph(3')-Ia*; *aph(3’’)-Ib*; *aph(6)-Id* | *dfrA7*; *sul1*; *sul2* | *oqxA*; *oqxB*; *fosA*; *catA1*; *tet(A)*; *erm(C)* |
| K021-H |  | 39 | IncFIB(K); IncFII(K); IncR; ColRNAI | K13:A-:B- | *bla*_TEM-1B_; *bla*_CTX-M-15_; *bla*_SHV-11_; *bla*_SCO-1_ | *aph(3')-Ia*; *aph(3’’)-Ib*; *aph(6)-Id* | *dfrA7*; *sul1*; *sul2* | *oqxA*; *oqxB*; *fosA*; *catA1*; *tet(A)*; *erm(C)* |
| K022-H |  | 107 | IncFIB(K); IncFII(K); IncR; ColRNAI | K5:A-:B- | *bla*_TEM-1B_; *bla*_CTX-M-15_; *bla*_SHV-1_ | *aac(3)-IId*; *aph(6)-Id* | *dfrA30*; *sul2* | *oqxA*; *oqxB*; *fosA*; *catA2*; *erm(C)* |
| K023-H |  | 14 | IncFIB(K); IncFII(K); IncR; ColRNAI | K5:A-:B- | *bla*_TEM-1B_; *bla*_CTX-M-15_; *bla*_SHV-28_ | *aac(3)-IId* | *dfrA14*; *dfrA30*; *sul2* | *oqxA*; *oqxB*; *fosB*; *mph(A)*; *catA2* |
| K024-H |  | 25 | IncFIB(K); IncFII(K); IncR; ColRNAI | nd | *bla*_TEM-1B_; *bla*_CTX-M-15_; *bla*_SHV-11_ | *aac(3)-IIa*; *aph(3’’)-Ib*; *aph(6)-Id* | *dfrA25*; *sul1*; *sul2* | *oqxA*; *oqxB*; q*nrB2*; *fosA*; *catA2*; *tet(D)* |
| K025-C |  | 394 | IncFIB(K); IncFII(K); IncR; ColRNAI | K5:A-:B- | *bla*_TEM-1B_; *bla*_CTX-M-15_; *bla*_SHV-11_ | *aac(3)-IId*; *aph(3’’)-Ib*; *aph(6)-Id* | *dfrA14*; *dfrA30*; *sul2* | *oqxA*; *oqxB*; *fosA*; *mph(A)*; *catA2*; *erm(B)* |
| K026-H |  | 336 | IncFIB(K); IncFII(K); IncR; ColRNAI | K5:A-:B- | *bla*_TEM-1B_; *bla*_CTX-M-15_; *bla*_SHV-11_ | *aac(3)-IId* | *dfrA14*; *dfrA30*; *sul2* | *oqxA*; *oqxB*; *fosA*; *mph(A)*; *catA2*; *erm(C)* |
| K028-H |  | 13 | IncFIB(K); IncFII(K); IncR; ColRNAI | K5:A-:B- | *bla*_TEM-1B_; *bla*_CTX-M-15_; *bla*_SHV-101_-like; *bla*_OXA-1_ | *aac(6')Ib-cr* | *dfrA30*; *sul2* | *oqxA*; *oqxB*; *qnrB2*; *fosA*; *catA2*; *catB4*; *tet(D)* |
| K029-H |  | 4 | IncFIB(K); IncFII(K); IncR; ColRNAI | K5:A-:B- | *bla*_TEM-1B_; *bla*_CTX-M-15_; *bla*_SHV-11_-like | *aph(3')-Ia*; *aph(3’’)-Ib*; *aph(6)-Id* | *dfrA14*; *dfrA30*; *sul2* | *oqxA*; *oqxB*; *fosA*; *mph(A)*; *tet(A)* |
| K030-H |  | 391 | IncFIB(K); IncFII(K); IncR; ColRNAI | K5:A-:B- | *bla*_TEM-1B_; *bla*_CTX-M-15_; *bla*_SHV-11_ | *aac(3)-IId* | *dfrA14*; *dfrA30* | *oqxA*; *oqxB*; *fosA*; *mph(A)* |
| K031-H |  | 14 | IncFIB(K); IncFII(K); IncR; ColRNAI | K5:A-:B- | *bla*_TEM-1B_; *bla*_CTX-M-15_; *bla*_SHV-28_ | *aac(3)-IId* | *dfrA14*; *dfrA30*; *sul2* | *oqxA*; *oqxB*; *fosA*; *mph(A)*; *catA2* |
| K032-H |  | 1726 | IncFIB(K); IncFII(K); IncR; ColRNAI | K5:A-:B- | *bla*_TEM-1B_; *bla*_CTX-M-15_; *bla*_SHV-27_ | *aac(3)-IId* | *dfrA30*; *sul2* | *oqxA*; *oqxB*; *fosA*; *catA2* |
| K033-H |  | 76 | IncFIB(K); IncFII(K); IncR; ColRNAI | K5:A-:B- | *bla*_TEM-1B_; *bla*_CTX-M-15_; *bla*_SHV-1_ | *aac(3)-IId* | *dfrA30*; *sul2* | *oqxA*; *oqxB*; *fosA* |
| K034-H |  | 3403 | IncFIB(K); IncFII(K); IncR; ColRNAI | K5:A-:B- | *bla*_TEM-1B_; *bla*_CTX-M-15_; *bla*_SHV-11_ | *aac(3)-IId* | *dfrA30*; *sul2* | *oqxA*; *oqxB*; *fosA*; *catA2* |
| K035-H |  | 3403 | IncFIB(K); IncFII(K); IncR; ColRNAI | K5:A-:B- | *bla*_TEM-1B_; *bla*_CTX-M-15_; *bla*_SHV-11_ | *aac(3)-IId* | *dfrA30*; *sul2* | *oqxA*; *oqxB*; *fosA*; *catA2* |
| K036-H |  | 834 | IncFIB(K); IncFII(K); IncR; ColRNAI | K5:A-:B- | *bla*_TEM-1B_; *bla*_CTX-M-15_; *bla*_SHV-11_ | *aac(3)-IId* | *dfrA14*; *dfrA30*; *sul2* | *oqxA*; *oqxB*; *fosA5*; *mph(A)*; *catA2* |
| K037-H |  | 391 | IncFIB(K); IncFII(K); IncR; ColRNAI | K5:A-:B- | *bla*_TEM-1B_; *bla*_CTX-M-15_; *bla*_SHV-11_ | *aac(3)-IId*; *aph(3’)-Ia*; aph*(3’’)-Ib*; *aph(6)-Id* | *dfrA14*; *dfrA30*; *sul2* | *oqxA*; *oqxB*; *fosA*; *mph(A)*; *catA2* |
| K038-H |  | 394 | IncFIB(K); IncFII(K); IncR; ColRNAI | K7:A-:B- | *bla*_TEM-1B_; *bla*_CTX-M-15_; *bla*_SHV-11_; *bla*_OXA-1_ | *aac(6')Ib-cr*; *aac(3)-IIa*; *aph(3’’)-Ib*; *aph(6)-Id* | *dfrA14*; *sul2* | *oqxA*; *oqxB*; *fosA* |
| K039-H |  | 403 | IncFIB(K); IncFII(K); IncR; ColRNAI | K5:A-:B- | *bla*_TEM-1B_; *bla*_CTX-M-15_; *bla*_SHV-36_-like | *aac(3)-IId* | *dfrA30*; *sul2* | *oqxA*; *oqxB*; *fosA* |
| K040-H |  | 788 | IncFIB(K); IncFII(K); IncR; ColRNAI | K7:A-:B- | *bla*_TEM-1B_; *bla*_CTX-M-15_; *bla*_SHV-52_; *bla*_OXA-1_ | *aac(6')Ib-cr*; *aac(3)-IIa*; *aph(3’’)-Ib*; *aph(6)-Id* | *dfrA14*; *sul2* | *oqxA*; *oqxB*; *qnrB1*; *fosA*; *tet(A)* |
| K041-H |  | 336 | IncFIB(K); IncFII(K); IncR; ColRNAI | K8:A-:B- | *bla*_TEM-1B_; *bla*_CTX-M-15_; *bla*_SHV-11_; *bla*_OXA-1_ | *aac(6')Ib-cr*; *aph(3’)-Ia*; *aph(3’’)-Ib*; *aph(6)-Id* | *dfrA14*; *sul2* | *oqxA*; *oqxB*; *qnrB1*; *fosA* |
| K042-H |  | 133 | IncFIB(K); IncFII(K); IncR; ColRNAI | K2:A-:B- | *bla*_TEM-1B_; *bla*_CTX-M-15_; *bla*_SHV-75_; *bla*_OXA-1_ | *aac(6')Ib-cr*; *aac(3)-IIa*; *aph(3’’)-Ib*; *aph(6)-Id* | *dfrA14*; *sul2* | *oqxA*; *oqxB*; *qnrB1* |
| K043-H |  | 252 | IncFIB(K); IncFII(K); IncR; ColRNAI | K5:A-:B- | *bla*_TEM-1B_; *bla*_CTX-M-15_; *bla*_SHV-1_ | *aac(3)-IId* | *dfrA30*; *sul2* | *oqxA*; *oqxB*; *fosA*; *catA2* |
| K044-H |  | 14 | IncFIB(K); IncFII(K); IncR; ColRNAI | K5:A-:B- | *bla*_TEM-1B_; *bla*_CTX-M-15_; *bla*_SHV-28_ | *aac(3)-IId* | *dfrA30*; *sul2* | *oqxA*; *oqxB*; *fosA*; *catA2* |
| K045-H |  | 30 | IncFIB(K); IncFII(K); IncR; ColRNAI | K5:A-:B- | *bla*_TEM-1B_; *bla*_CTX-M-15_; *bla*_SHV-11_ | *aac(3)-IId*; *aadA2* | *dfrA12*; *dfrA30*; *sul1*; *sul2* | *oqxA*; *oqxB*; *fosA*; *mph(A)*; *catA2* |
| K046-H |  | 336 | IncFIB(K); IncFII(K); IncR; ColRNAI | K5:A-:B- | *bla*_TEM-1B_; *bla*_CTX-M-15_; *bla*_SHV-11_ | *aac(3)-IId*; *aph(3’)-Ia*; aph*(3’’)-Ib*; *aph(6)-Id* | *dfrA14*; *dfrA30* | *oqxA*; *oqxB*; *fosA*; *mph(A)*; *catA2* |
| K047-H |  | 471 | IncFIB(K); IncFII(K); IncR; ColRNAI | K7:A-:B- | *bla*_TEM-1B_; *bla*_CTX-M-15_; *bla*_SHV-108_; *bla*_OXA-1_ | *aac(6')Ib-cr*; *aac(3)-IIa*; *aph(3’’)-Ib*; *aph(6)-Id* | *dfrA14*; *sul2* | *oqxA*; *oqxB*; *qnrB1*; *fosA*; *tet(A)* |
| K049-H |  | 1800 | IncFIB(K); IncFII(K); IncR; ColRNAI | K15:A-:B- | *bla*_TEM-1B_; *bla*_CTX-M-15_; *bla*_SHV-11_; *bla*_OXA-1_ | *aac(6')Ib-cr*; *aac(3)-IIa*; *aph(3’’)-Ib*; *aph(6)-Id* | *dfrA14*; *sul2* | *oqxA*; *oqxB*; *fosA* |
| K050-H |  | 30 | IncFIB(K); IncFII(K); IncR; ColRNAI | K5:A-:B- | *bla*_TEM-1B_; *bla*_CTX-M-15_; *bla*_SHV-11_ | *aac(3)-IId*; *aadA2* | *dfrA12*; *dfrA30*; *sul1*; *sul2* | *oqxA*; *oqxB*; *fosA*; *mph(A*); *catA2* |
| K051-H |  | 3405 | IncFIB(K); IncFII(K); IncR; ColRNAI | K5:A-:B- | *bla*_TEM-1B_; *bla*_CTX-M-15_; *bla*_SHV-44_-like | *aph(3')-Ia*; *aph(3’’)-Ib*; *aph(6)-Id*; *aac(3)-IId* | *dfrA7*; *dfrA30*; *sul1*; *sul2*-like | *oqxA*; *oqxB*; *fosA*; *catA2*; *tet(D)* |
| K052-H |  | 405 | IncFIB(K); IncFII(K); IncR; ColRNAI | K5:A-:B- | *bla*_TEM-1B_; *bla*_CTX-M-15_; *bla*_SHV-76_ | *aac(3)-IId* | *dfrA30*; *sul2* | *oqxA*; *oqxB*; *fosA*; *catA2* |
| K053-H |  | 6 | IncFIB(K); IncFII(K); IncR; ColRNAI | K5:A-:B- | *bla*_TEM-1B_; *bla*_CTX-M-15_; *bla*_SHV-1_ | *aac(3)-IId* | *dfrA30*; *sul2* | *oqxA*; *oqxB*; *fosA* |
| K054-H |  | 37 | IncFIB(K); IncFII(K); IncR; ColRNAI | K5:A-:B- | *bla*_TEM-1B_; *bla*_CTX-M-15_; *bla*_SHV-11_ | *aac(3)-IId* | *dfrA30* | *oqxA*; *oqxB*; *fosA* |
| K055-H |  | 3438 | IncFIB(K); IncFII(K); IncR; ColRNAI | K5:A-:B- | *bla*_TEM-1B_; *bla*_CTX-M-15_; *bla*_SHV-1_ | *aac(3)-IId* | *dfrA30*; *sul2* | *oqxA*; *oqxB*; *fosA*; *catA2* |
| K056-H |  | 17 | IncFIB(K); IncFII(K); IncR; ColRNAI | K5:A-:B- | *bla*_TEM-1B_; *bla*_CTX-M-15_; *bla*_SHV-11_ | *aac(3)-IId* | *dfrA7*; *dfrA30*; *sul1*; *sul2* | *oqxA*; *oqxB*; *fosA*; *tet(D)* |
| K057-H |  | 323 | IncFIB(K); IncFII(K); IncR; ColRNAI | K5:A-:B- | *bla*_TEM-1B_; *bla*_CTX-M-15_; *bla*_SHV-99_-like | *aac(3)-IId* | *dfrA30*; *sul2* | *oqxA*; *oqxB*; *fosA*; *catA2* |
| K058-H |  | 323 | IncFIB(K); IncFII(K); IncR; ColRNAI | K5:A-:B- | *bla*_TEM-1B_; *bla*_CTX-M-15_; *bla*_SHV-99_-like | *aac(3)-IId* | *dfrA30*; *sul2* | *oqxA*; *oqxB*; *fosA*; *catA2* |
| K059-H |  | 268 | IncFIB(K); IncFII(K); IncR; ColRNAI | K12:A-:B- | *bla*_TEM-1B_; *bla*_CTX-M-15_; *bla*_SHV-11_; *bla*_OXA-1_ | *aac(6')Ib-cr*; *aac(3)-IIa*; *aph(3’’)-Ib*; *aph(6)-Id* | *dfrA14*; *sul2* | *oqxA*; *oqxB*; *qnrB1*; *fosA5*; *tet(A)* |
| K060-H |  | 15 | IncFIB(K); IncFII(K); IncR; ColRNAI | K5:A-:B- | *bla*_TEM-1B_; *bla*_CTX-M-15_; *bla*_SHV-28_ | *aac(3)-IId* | *dfrB4*; *dfrA30*; *sul1*; *sul2* | *oqxA*; *oqxB*; *qepA2*; *fosA*;*catA2*; *tet(A)* |
| K061-H |  | 405 | IncFIB(K); IncFII(K); IncR; ColRNAI | K5:A-:B- | *bla*_TEM-1B_; *bla*_CTX-M-15_; *bla*_SHV-76_ | *aac(3)-IId* | *dfrA30*; *sul2* | *oqxA*; *oqxB*; *fosA*;*catA2* |
| K062-H |  | 323 | IncFIB(K); IncFII(K); IncR; ColRNAI | K5:A-:B- | *bla*_TEM-1B_; *bla*_CTX-M-15_; *bla*_SHV-99_-like | *aac(3)-IId* | *dfrA30*; *sul2* | *oqxA*; *oqxB*; *fosA*;*catA2* |
| K063-H |  | 471 | IncFIB(K); IncFII(K); IncR; ColRNAI | K7:A-:B- | *bla*_TEM-1B_; *bla*_CTX-M-15_; *bla*_SHV-108_; *bla*_OXA-1_ | *aac(6')Ib-cr*; *aac(3)-IIa*; *aph(3’’)-Ib*; *aph(6)-Id* | *dfrA14*; *sul2* | *oqxA*; *oqxB*; *qnrB1*; *fosA* |
| K064-H |  | 372 | IncFIB(K); IncFII(K); IncR; ColRNAI | K5:A-:B- | *bla*_TEM-1B_; *bla*_CTX-M-15_; *bla*_SHV-26_ | *aac(3)-IId* | *dfrA30*; *sul2* | *oqxA*; *oqxB*; *fosA*;*catA2* |
| K065-H |  | 3405 | IncFIB(K); IncFII(K); IncR; ColRNAI | K5:A-:B- | *bla*_TEM-1B_; *bla*_CTX-M-15_; *bla*_SHV-1_ | *aph(3')-Ia*; *aph(3’’)-Ib*; *aph(6)-Id*; *aac(3)-IId* | *dfrA7*; *dfrA30*; *sul1* | *oqxA*; *oqxB*; *fosA*; *catA2*; *tet(D)* |
| K066-H |  | 3437 | IncFIB(K); IncFII(K); IncR; ColRNAI | K5:A-:B- | *bla*_TEM-1B_; *bla*_CTX-M-15_; *bla*_SHV-11_ | *aac(3)-IId* | *dfrA30*; *sul2* | *oqxA*; *oqxB*; *fosA*; *catA2* |
| K067-H |  | 1726 | IncFIB(K); IncFII(K); IncR; ColRNAI | K5:A-:B- | *bla*_TEM-1B_; *bla*_CTX-M-15_; *bla*_SHV-27_ | *aac(3)-IId* | *dfrA30*; *sul2* | *oqxA*; *oqxB*; *fosA* |
| K068-H |  | 15 | IncFIB(K); IncFII(K); IncR; ColRNAI | K5:A-:B- | *bla*_TEM-1B_; *bla*_CTX-M-15_; *bla*_SHV-27_ | *aac(3)-IId* | *dfrA30*; *sul2* | *oqxA*; *oqxB*; *fosA*; *catA2* |
| K069-H |  | 3433 | IncFIB(K); IncFII(K); IncR; ColRNAI | K5:A-:B- | *bla*_TEM-1B_; *bla*_CTX-M-15_; *bla*_SHV-11_ | *aac(3)-IId* | *dfrA30*; *sul2* | *oqxA*; *oqxB*; *fosA* |
| K070-H |  | 13 | IncFIB(K); IncFII(K); IncR; ColRNAI | K5:A-:B- | *bla*_TEM-1B_; *bla*_CTX-M-15_; *bla*_SHV-101_-like | *aac(3)-IId* | *dfrA30*; *sul2* | *oqxA*; *oqxB*; *fosA*; *catA2* |
| K072-H |  | 3404 | IncFIB(K); IncFII(K); IncR; ColRNAI | K5:A-:B- | *bla*_TEM-1B_; *bla*_CTX-M-15_; *bla*_SHV-27_ | *aac(3)-IId* | *dfrA30*; *sul2* | *oqxA*; *oqxB*; *fosA*; *catA2* |
| K073-H |  | 29 | IncFIB(K); IncFII(K); IncR; ColRNAI | K7:A-:B- | *bla*_TEM-1B_; *bla*_CTX-M-15_; *bla*_SHV-83_; *bla*_OXA-1_ | *aac(6')Ib-cr*; *aac(3)-IIa*; *aph(3’’)-Ib*; *aph(6)-Id* | *dfrA14*; *sul2* | *oqxA*; *oqxB*; *qnrB1*; *fosA*; *tet(A)* |
| K074-H |  | 45 | IncFIB(K); IncFII(K); IncR; ColRNAI | K5:A-:B- | *bla*_TEM-1B_; *bla*_CTX-M-15_; *bla*_SHV-1_; *bla*_SHV-148_-like | *aac(3)-IId* | *dfrA15*; *dfrA30*; *sul1*; *sul2* | *oqxA*; *oqxB*; *fosA*; *catA1* |
| K075-H |  | 661 | IncFIB(K); IncFII(K); IncR; ColRNAI | K5:A-:B- | *bla*_TEM-1B_; *bla*_CTX-M-15_; *bla*_SHV-27_ | *aac(3)-IId* | *dfrA30*; *sul2* | *oqxA*; *oqxB*; *fosA*; *catA2* |
| K076-H |  | 397 | IncFIB(K); IncFII(K); IncR; ColRNAI | K5:A-:B- | *bla*_CTX-M-15_; *bla*_SHV-1_ |  | *dfrA30* | *oqxA*; *oqxB*; *fosA* |
| K077-H |  | 1800 | IncFIB(K); IncFII(K); IncR; ColRNAI | K15:A-:B- | *bla*_TEM-1B_; *bla*_CTX-M-15_; *bla*_SHV-11_; *bla*_OXA-1_ | *aac(6')Ib-cr*; *aac(3)-IIa*; *aph(3’’)-Ib*; *aph(6)-Id* | *dfrA14*; *sul2* | *oqxA*; *oqxB*; *fosA* |
| K078-H |  | 268 | IncFIB(K); IncFII(K); IncR; ColRNAI | K12:A-:B- | *bla*_TEM-1B_; *bla*_CTX-M-15_; *bla*_SHV-11_; *bla*_OXA-1_ | *aac(6')Ib-cr*; *aac(3)-IIa*; *aph(3’’)-Ib*; *aph(6)-Id* | *dfrA14*; *sul2* | *oqxA*; *oqxB*; *qnrB1*; *fosA5*; *tet(A)* |
| K079-H |  | 1552 | IncFIB(K); IncFII(K); IncR; ColRNAI | K5:A-:B- | *bla*_TEM-1B_; *bla*_CTX-M-15_; *bla*_SHV-62_ |  | *dfrA30*; *sul2* | *oqxA*; *oqxB*; *fosA*; *catA1* |
| K080-H |  | 17 | IncFIB(K); IncFII(K); IncR; ColRNAI | K5:A-:B- | *bla*_TEM-1B_; *bla*_CTX-M-15_; *bla*_SHV-11_ | *aac(3)-IId* | *dfrA30*; *sul2* | *oqxA*; *oqxB*; *fosA*; *catA2* |
| K081-H |  | 629 | IncFIB(K); IncFII(K); IncR; ColRNAI | K5:A-:B- | *bla*_TEM-1B_; *bla*_CTX-M-15_; *bla*_SHV-11_; *bla*_OXA-1_ | *aac(6')Ib-cr*; *aph(3’’)-Ib*; *aph(6)-Id* | *dfrA25*; *dfrA30*; *sul1*; *sul2* | *oqxA*; *oqxB*; *qnrB2*; *fosA*; *catB4* |
| K082-H |  | 13 | IncFIB(K); IncFII(K); IncR; ColRNAI | K5:A-:B- | *bla*_TEM-1B_; *bla*_CTX-M-15_; *bla*_SHV-101_-like | *aac(3)-IId* | *dfrA30*; *sul2* | *oqxA*; *oqxB*; *fosA*; *catA2* |
| K083-H |  | 661 | IncFIB(K); IncFII(K); IncR; ColRNAI | K5:A-:B- | *bla*_TEM-1B_; *bla*_CTX-M-15_; *bla*_SHV-27_ | *aac(3)-IId* | *dfrA30*; *sul2* | *oqxA*; *oqxB*; *fosA*; *catA2* |
| K084-H |  | 336 | IncFIB(K); IncFII(K); IncR; ColRNAI | K5:A-:B- | *bla*_TEM-1B_; *bla*_CTX-M-15_; *bla*_SHV-11_ | *aac(3)-IId* | *dfrA14*; *dfrA30*; *sul2* | *oqxA*; *oqxB*; *fosA*; *mph(A)*; *catA2* |
| K085-H |  | 336 | IncFIB(K); IncFII(K); IncR; ColRNAI | K5:A-:B- | *bla*_TEM-1B_; *bla*_CTX-M-15_; *bla*_SHV-11_ | *aac(3)-IId* | *dfrA14*; *dfrA30*; *sul2* | *oqxA*; *oqxB*; *fosA*; *mph(A)*; *catA2* |
| K086-H |  | 111 | IncFIB(K); IncFII(K); IncR; ColRNAI | K5:A-:B- | *bla*_TEM-1B_; *bla*_CTX-M-15_; *bla*_SHV-11_ | *aac(3)-IId* | *dfrA30*; *sul2* | *oqxA*; *oqxB*; *fosA*; *catA2* |
| K087-H |  | 336 | IncFIB(K); IncFII(K); IncR; ColRNAI | K5:A-:B- | *bla*_TEM-1B_; *bla*_CTX-M-15_; *bla*_SHV-11_ | *aac(3)-IId* | *dfrA14*; *dfrA30*; *sul2* | *oqxA*; *oqxB*; *fosA*; *mph(A)*; *catA2* |
| K088-H |  | 194 | IncFIB(K); IncFII(K); IncR; ColRNAI | K5:A-:B- | *bla*_TEM-1B_; *bla*_CTX-M-15_; *bla*_SHV-11_ | *aac(3)-IId*¸ *aph(6)-Id* | *dfrA14*; *dfrA30*; *sul2* | *oqxA*; *oqxB*; *fosA*; *catA1*; *catA2*; *tet(D)* |
| K089-H |  | 1552 | IncFIB(K); IncFII(K); IncR; ColRNAI | K1:A-:B- | *bla*_CTX-M-15_; *bla*_SHV-62_-like |  | *dfrA30*; *sul2* | *oqxA*; *oqxB*; *fosA*; *catA1* |
| K090-H |  | 1552 | IncFIB(K); IncFII(K); IncR; ColRNAI | K1:A-:B- | *bla*_TEM-1B_; *bla*_CTX-M-15_; *bla*_SHV-62_-like | *aac(3)-IId* | *dfrA30*; *sul2* | *oqxA*; *oqxB*; *fosA*; *catA1* |
| K091-H |  | 37 | IncFIB(K); IncFII(K); IncR; ColRNAI | K1:A-:B- | *bla*_TEM-1B_; *bla*_CTX-M-15_; *bla*_SHV-26_; *bla*_OXA-1_ | *aac(6')Ib-cr*; *aac(3)-IIa*; *aph(3’)-Ia*; *aph(3’’)-Ib*; *aph(6)-Id*; *aadA4* | *dfrA21*; *sul2* | *oqxA*; *oqxB*; *fosA*; *catA1*; *tet(A); tet(D)* |
| K092-H |  | 20 | IncFIB(K); IncFII(K); IncR; ColRNAI | F-:A13-:B- | *bla*_TEM-1B_; *bla*_CTX-M-15_; *bla*_SHV-83_; *bla*_OXA-1_ | *aac(6')Ib-cr*; *aac(3)-IId*; *aph(3’’)-Ib*; *aph(6)-Id* | *dfrA14*; *dfrA25*; *sul1*; *sul2* | *oqxA*; *oqxB*; *qnrB2*; *fosA*; *catA2*; *tet(D)* |
| K093-H |  | 3438 | IncFIB(K); IncFII(K); IncR; ColRNAI | K5:A-:B- | *bla*_TEM-1B_; *bla*_CTX-M-15_; *bla*_SHV-1_ | *aac(3)-IId* | *dfrA30*; *sul2* | *oqxA*; *oqxB*; *fosA*; *catA2* |
| K095-H |  | 3403 | IncFIB(K); IncFII(K); IncR; ColRNAI | K5:A-:B- | *bla*_TEM-1B_; *bla*_CTX-M-15_; *bla*_SHV-11_ | *aac(3)-IId* | *dfrA30*; *sul2* | *oqxA*; *oqxB*; *fosA*; *catA2* |
| K096-H |  | 76 | IncFIB(K); IncFII(K); IncR; ColRNAI | K5:A-:B- | *bla*_TEM-1B_; *bla*_CTX-M-15_; *bla*_SHV-1_ | *aac(3)-IId* | *dfrA30*; *sul2* | *oqxA*; *oqxB*; *fosA* |
| K097-H |  | 39 | IncFIB(K); IncFII(K); IncR; ColRNAI | K13:A-:B- | *bla*_TEM-1B_; *bla*_CTX-M-15_; *bla*_SHV-11_ | *aph(3’)-Ia*; *aph(3’’)-Ib*; *aph(6)-Id* | *dfrA7*; *sul1*; *sul2* | *oqxA*; *oqxB*; *fosA*; *catA1*; *tet(A)* |
| K098-H |  | 45 | IncFIB(K); IncFII(K); IncR; ColRNAI | K5:A-:B- | *bla*_TEM-1B_; *bla*_CTX-M-15_; *bla*_SHV-1_ | *aac(3)-IId* | *dfrA30*; *sul2* | *oqxA*; *oqxB*; *fosA*; *catA2* |
| K099-H |  | 3434 | IncFIB(K); IncFII(K); IncR; ColRNAI | K12:A21*:B- | *bla*_TEM-1B_; *bla*_CTX-M-15_; *bla*_SHV-83_; *bla*_OXA-1_ | *aac(6')Ib-cr*; *aac(3)-IIa*; *aph(3’’)-Ib*; *aph(6)-Id* | *dfrA14*; *sul2* | *oqxA*; *oqxB*; *qnrB1*; *fosA*; *tet(A)* |
| K100-C |  | 3403 | IncFIB(K); IncFII(K); IncR; ColRNAI | K5:A-:B- | *bla*_TEM-1B_; *bla*_CTX-M-15_; *bla*_SHV-11_ | *aac(3)-IId* | *dfrA30*; *sul2* | *oqxA*; *oqxB*; *fosA*; *catA2* |
| K101-C |  | 834 | IncFIB(K); IncFII(K); IncR; ColRNAI | K5:A-:B- | *bla*_TEM-1B_; *bla*_CTX-M-15_; *bla*_SHV-11_ | *aac(3)-IId* | *dfrA14*; *dfrA30*; *sul2* | *oqxA*; *oqxB*; *fosA5*; *mph(A)*; *catA2* |
| K102-C |  | 397 | IncFIB(K); IncFII(K); IncR; ColRNAI | K5:A-:B- | *bla*_TEM-1B_; *bla*_CTX-M-15_; *bla*_SHV-1_ | *aac(3)-IId* | *dfrA30*; *sul2* | *oqxA*; *oqxB*; *fosA* |
| K103-C |  | 323 | IncFIB(K); IncFII(K); IncR; ColRNAI | K5:A-:B- | *bla*_TEM-1B_; *bla*_CTX-M-15_; *bla*_SHV-99_-like | *aac(3)-IId* | *dfrA15*; *dfrA30*; *sul1*; *sul2* | *oqxA*; *oqxB*; *fosA* |
| K104-C |  | 14 | IncFIB(K); IncFII(K); IncR; ColRNAI | K1:A-:B- | *bla*_TEM-1B_; *bla*_CTX-M-15_; *bla*_SHV-28_ | *aac(3)-IId*; *aadA1* | *dfrA30*; *sul1*; *sul2* | *oqxA*; *oqxB*; *fosA*; *catA2*; *tet(D)* |
| K105-C |  | 3435 | IncFIB(K); IncFII(K); IncR; ColRNAI | K5:A-:B- | *bla*_TEM-1B_; *bla*_CTX-M-15_; *bla*_SHV-38_-like | *aac(3)-IId* | *dfrA30*; *sul2* | *oqxA*; *oqxB*; *fosA* |
| K106-H |  | 39 | IncFIB(K); IncFII(K); IncR; ColRNAI | K2:A-:B- | *bla*_TEM-1B_; *bla*_CTX-M-15_; *bla*_SHV-12_; *bla*_SCO-1_ | *aph(3’)-Ia*; *aph(3’’)-Ib*; *aph(6)-Id* | *dfrA7*; *sul1*; *sul2* | *oqxA*; *oqxB*; *fosA*; *catA1*; *tet(A)* |
| K107-H |  | 48 | IncFIB(K); IncFII(K); IncR; ColRNAI | K5:A-:B- | *bla*_TEM-1B_; *bla*_CTX-M-15_; *bla*_SHV-11_ | *aac(3)-IId* | *dfrA30*; *sul2* | *oqxA*; *oqxB*; *catA2* |
| K108-H |  | 2816 | IncFIB(K); IncFII(K); IncR; ColRNAI | K5:A-:B- | *bla*_CTX-M-15_; *bla*_SHV-11_ |  | *dfrA30*; *sul2* | *oqxA*; *oqxB*; *fosA*; *catA2* |
| K109-H |  | 189 | IncFIB(K); IncFII(K); IncR; ColRNAI | K5:A-:B- | *bla*_TEM-1B_; *bla*_CTX-M-15_; *bla*_SHV-80_ | *aac(3)-IId*; *aadA2* | *dfrA12*; *dfrA30*; *sul1*; *sul2* | *oqxA*; *oqxB*; *fosA*; *mph(A)*; *catA2*; *tet(A)* |
| K110-H |  | 39 | IncFIB(K); IncFII(K); IncR; ColRNAI | K13:A-:B- | *bla*_TEM-1B_; *bla*_CTX-M-15_; *bla*_SHV-12_; *bla*_SCO-1_ | *aph(3’)-Ia*; *aph(3’’)-Ib*; *aph(6)-Id* | *dfrA7*; *sul1*; *sul2* | *oqxA*; *oqxB*; *fosA*; *catA1*; *tet(A)* |
| K111-H |  | 133 | IncFIB(K); IncFII(K); IncR; ColRNAI | K7:A-:B- | *bla*_TEM-1B_; *bla*_CTX-M-15_; *bla*_SHV-75_; *bla*_OXA-1_-like | *aac(6')Ib-cr*; *aac(3)-IIa*; *aph(3’’)-Ib*; *aph(6)-Id* | *dfrA14*; *sul2* | *oqxA*; *oqxB*; *fosA*; *catA2* |
| K112-H |  | 280 | IncFIB(K); IncFII(K); IncR; ColRNAI | K5:A-:B- | *bla*_TEM-1B_; *bla*_CTX-M-15_; *bla*_SHV-27_ | *aac(3)-IId* | *dfrA30*; *sul2* | *oqxA*; *oqxB*; *catA2* |
| K113-H |  | 391 | IncFIB(K); IncFII(K); IncR; ColRNAI | K5:A-:B- | *bla*_TEM-1B_; *bla*_CTX-M-15_; *bla*_SHV-11_ | *aac(3)-IId*; *aph(3’)-Ia*; *aph(3’’)-Ib*; *aph(6)-Id* | *dfrA14*; *dfrA30* | *oqxA*; *oqxB*; *fosA*; *mph(A)*; *catA2* |
| K114-H |  | 834 | IncFIB(K); IncFII(K); IncR; ColRNAI | K5:A-:B- | *bla*_TEM-1B_; *bla*_CTX-M-15_; *bla*_SHV-11_ | *aac(3)-IId* | *dfrA30*; *sul2* | *oqxA*; *oqxB*; *fosA5*; *catA2* |
| K115-H |  | 348 | IncFIB(K); IncFII(K); IncR; ColRNAI | K5:A-:B- | *bla*_TEM-1B_; *bla*_CTX-M-15_; *bla*_SHV-11_ | *aac(3)-IId*; *aph(3’)-Ia*; *aph(3’’)-Ib*; *aph(6)-Id* | *dfrA14*; *dfrA30* | *oqxA*; *oqxB*; *fosA*; *mph(A)* |
| K116-H |  | 17 | IncFIB(K); IncFII(K); IncR; ColRNAI | nd | *bla*_CTX-M-15_; *bla*_SHV-11_; *bla*_OXA-1_ | *aac(6')Ib-cr*; *aac(3)-IIa*; *aph(3’)-Ia*; *aadA2* | *dfrA12*; *sul1*; *sul2* | *oqxA*; *oqxB*; *fosA*; *mph(A)*; *catA2* |
| K117-H |  | 45 | IncFIB(K); IncFII(K); IncR; ColRNAI | K1:A-:B- | *bla*_TEM-1B_; *bla*_CTX-M-15_; *bla*_SHV-1_; *bla*_OXA-1_ | *aac(6')Ib-cr*; *aac(3)-IIa*; *aph(3’’)-Ib*; *aph(6)-Id* | *dfrA14*; *dfrA15*; *sul1*; *sul2* | *oqxA*; *oqxB*; *fosA*; *catA1* |
| K118-H |  | 313 | IncFIB(K); IncFII(K); IncR; ColRNAI | K5:A-:B- | *bla*_TEM-1B_; *bla*_CTX-M-15_; *bla*_SHV-1_-like | *aac(3)-IId* | *dfrA30*; *sul2* | *oqxA*; *oqxB*; *fosA*; *catA2* |
| K119-H |  | 1411 | IncFIB(K); IncFII(K); IncR; ColRNAI | K5:A-:B- | *bla*_TEM-1B_; *bla*_CTX-M-15_; *bla*_SHV-11_ | *aac(3)-IId*; *aph(3’’)-Ib*; *aph(6)-Id* | *dfrA5*; *dfrA30*; *sul2* | *oqxA*; *oqxB*; *fosA*; *catA1*; *catA2* |
| K120-H |  | 30 | IncFIB(K); IncFII(K); IncR; ColRNAI | K5:A-:B- | *bla*_TEM-1B_; *bla*_CTX-M-15_; *bla*_SHV-11_ | *aac(3)-IId*; *aadA2* | *dfrA12*; *dfrA30*; *sul1*; *sul2* | *oqxA*; *oqxB*; *fosA*; *mph(A)*; *catA2* |
| K121-H |  | 471 | IncFIB(K); IncFII(K); IncR; ColRNAI | K7:A-:B- | *bla*_TEM-1B_; *bla*_CTX-M-15_; *bla*_SHV-108_-like; *bla*_OXA-1_ | *aac(6')Ib-cr*; *aac(3)-IIa*; *aph(3’’)-Ib*; *aph(6)-Id* | *dfrA14*; *sul2* | *oqxA*; *oqxB*; *qnrB1*; *fosA* |
| K122-H |  | 39 | IncFIB(K); IncFII(K); IncR; ColRNAI | K5:A-:B- | *bla*_TEM-1B_; *bla*_CTX-M-15_; *bla*_SHV-1_-like | *aac(3)-IId*; *aph(3’’)-Ib*; *aph(6)-Id* | *dfrA30*; *sul2* | *oqxA*; *oqxB*; *fosA* |
| K123-H |  | 815 | IncFIB(K); IncFII(K); IncR; ColRNAI | K5:A-:B- | *bla*_TEM-1B_; *bla*_CTX-M-15_; *bla*_SHV-11_ | *aac(3)-IId*; *aph(3’’)-Ib*; *aph(6)-Id* | *dfrA30*; *sul2* | *oqxA*; *oqxB*; *fosA*; *catA2*; *tet(A)* |
| K124-H |  | 1552 | IncFIB(K); IncFII(K); IncR; ColRNAI | K1:A-:B- | *bla*_TEM-1B_; *bla*_CTX-M-15_; *bla*_SHV-62_-like | *aac(3)-IId* | *dfrA30*; *sul2* | *oqxA*; *oqxB*; *fosA*; *catA1* |
| K125-H |  | 39 | IncFIB(K); IncFII(K); IncR; ColRNAI | K5:A-:B- | *bla*_TEM-1B_; *bla*_CTX-M-15_; *bla*_SHV-11_ | *aac(3)-IId*; *aph(3’’)-Ib*; *aph(6)-Id*; *aadA2* | *dfrA12*; *dfrA30*; *sul1*; *sul2* | *oqxA*; *oqxB*; *fosA*; *mph(A)*; *tet(A)* |
| K126-H |  | 336 | IncFIB(K); IncFII(K); IncR; ColRNAI | K5:A-:B- | *bla*_TEM-1B_; *bla*_CTX-M-15_; *bla*_SHV-11_ | *aac(3)-IId* | *dfrA30*; *sul2* | *oqxA*; *oqxB*; *fosA*; *catA2* |
| K127-H |  | 280 | IncFIB(K); IncFII(K); IncR; ColRNAI | K5:A-:B- | *bla*_TEM-1B_; *bla*_CTX-M-15_; *bla*_SHV-27_ | *aac(3)-IId* | *dfrA30*; *sul2* | *oqxA*; *oqxB*; *fosA*; *catA2* |
| K128-H |  | 1798 | IncFIB(K); IncFII(K); IncR; ColRNAI | K7:A-:B- | *bla*_TEM-1B_; *bla*_CTX-M-15_; *bla*_SHV-1_; *bla*_OXA-1_ | *aac(6')Ib-cr*; *aph(3’’)-Ib*; *aph(6)-Id* | *dfrA14*; *sul2* | *oqxA*; *oqxB*; *qnrB1*; *fosA*; *tet(A)* |
| K129-H |  | 307 | IncFIB(K); IncFII(K); IncR; ColRNAI | K7:A-:B- | *bla*_TEM-1B_; *bla*_CTX-M-15_; *bla*_SHV-28_; *bla*_OXA-1_ | *aac(6')Ib-cr*; *aph(3’’)-Ib*; *aph(6)-Id* | *dfrA14*; *sul2* | *oqxA*; *oqxB*; *qnrB1*; *fosA*; *tet(A)* |
| K130-H |  | 3403 | IncFIB(K); IncFII(K); IncR; ColRNAI | K5:A-:B- | *bla*_TEM-1B_; *bla*_CTX-M-15_; *bla*_SHV-11_ | *aac(3)-IId* | *dfrA30*; *sul2* | *oqxA*; *oqxB*; *fosA*; *catA2* |
| K131-H |  | 788 | IncFIB(K); IncFII(K); IncR; ColRNAI | K7:A-:B- | *bla*_TEM-1B_; *bla*_CTX-M-15_; *bla*_SHV-52_; *bla*_OXA-1_ | *aac(6')Ib-cr*; *aac(3)-IIa*; *aph(3’’)-Ib*; *aph(6)-Id* | *dfrA14*; *sul2* | *oqxA*; *oqxB*; *qnrB1*; *fosA*; *tet(A)* |
| K132-H |  | 336 | IncFIB(K); IncFII(K); IncR; ColRNAI | K5:A-:B- | *bla*_TEM-1B_; *bla*_CTX-M-15_; *bla*_SHV-11_ | *aac(3)-IId*; *aph(3’)-Ia* | *dfrA30*; *sul2* | *oqxA*; *oqxB*; *fosA*; *mph(A)* |
| K133-H |  | 1726 | IncFIB(K); IncFII(K); IncR; ColRNAI | K5:A-:B- | *bla*_TEM-1B_; *bla*_CTX-M-15_; *bla*_SHV-27_ | *aac(3)-IId* | *dfrA30*; *sul2* | *oqxA*; *oqxB*; *fosA*; *catA2* |
| K134-H |  | 391 | IncFIB(K); IncFII(K); IncR; ColRNAI | K5:A-:B- | *bla*_TEM-1B_; *bla*_CTX-M-15_; *bla*_SHV-11_ | *aac(3)-IId*; *aph(3’)-Ia* *aph(3’’)-Ib*; *aph(6)-Id* | *dfrA14*; *dfrA30* | *oqxA*; *oqxB*; *fosA*; *mph(A)*; *catA2* |

1. Sequence type determined by multi locus sequence typing. [↑](#footnote-ref-1)
2. If more than one IncFII(K)-type was detected, only K5 is shown in table. [↑](#footnote-ref-2)
3. Community [↑](#footnote-ref-3)
4. Hospitalized [↑](#footnote-ref-4)
5. Not determined (one or more loci do not match 100% to any previously registered pMLST allele). [↑](#footnote-ref-5)
