# Supplementary material for: Horizontal Plasmid Transfer among Klebsiella pneumoniae Isolates Is the Key Factor for Dissemination of Extended-Spectrum β-Lactamases among Children in Tanzania
Source: mSphere. 2020 Jul 15;5(4):e00428-20. doi: 10.1128/mSphere.00428-20 (PMC7364214; doi:10.1128/mSphere.00428-20)
Supplement: TABLE S2 [file mSphere.00428-20-st002.docx]

**TABLE S2** Characteristics of the 128 CTX-M-15 positive *K. pneumoniae* carriers

| **Characteristics** | **Carriers of CTX-M-15 positive *K. pneumoniae*** | | **Total study population^3^** | | **Prevalence of carriers^4^** |
| --- | --- | --- | --- | --- | --- |
| **Sex** | **N children** | **%** | **N children** | **%** | **%** |
| Male | 87 | 68.0 | 361 | 59.9 | 24.1 |
| Female | 41 | 32.0 | 242 | 40.1 | 16.9 |
| **Age** |  |  |  |  |  |
| ≤ 12 months | 102 | 79.7 | 372 | 61.7 | 27.4 |
| > 12 months | 26 | 20.3 | 231 | 38.3 | 11.3 |
| **Place of residence (district)** |  |  |  |  |  |
| Ilala | 51 | 39.8 | 289 | 47.9 | 17.7 |
| Kinondoni | 49 | 38.3 | 179 | 29.7 | 27.4 |
| Temeke | 28 | 21.9 | 135 | 22.4 | 20.7 |
| **Study group** |  |  |  |  |  |
| Community | 12 | 9.4 | 250 | 41.5 | 4.8 |
| Hospitalized | 116 | 90.6 | 353 | 58.5 | 32.9 |
| **HIV^1^** |  |  |  |  |  |
| Positive | 18 | 41.9 | 29 | 8.3 | 62.1 |
| Negative | 25 | 58.1 | 319 | 91.7 | 7.8 |
| **Use of antibiotics^2^** |  |  |  |  |  |
| No use | 57 | 44.5 | 378 | 62.7 | 15.1 |
| Used | 71 | 55.5 | 225 | 37.3 | 31.6 |

N: Total number of children; %: number of children in percentage.

^1^Children with known HIV status

^2^Use of antibiotics during the last 14 days prior to inclusion or on inclusion.

^3^In the previous published study including 603 children of whom 207 were ESBL-carriers.

^4^Prevalence of CTX-M-15 positive *K. pneumoniae* carriers in the total study population (%)
