# Supplementary material for: Horizontal Plasmid Transfer among Klebsiella pneumoniae Isolates Is the Key Factor for Dissemination of Extended-Spectrum β-Lactamases among Children in Tanzania
Source: mSphere. 2020 Jul 15;5(4):e00428-20. doi: 10.1128/mSphere.00428-20 (PMC7364214; doi:10.1128/mSphere.00428-20)
Supplement: TABLE S3 [file mSphere.00428-20-st003.docx]

**TABLE S3** MICs for *K. pneumoniae* strains with completed genomes

|  |  | **MIC (mg/L) of^[[1]](#footnote-1)^** | | | | | | | | | | | | | | |
| --- | --- | --- | --- | --- | --- | --- | --- | --- | --- | --- | --- | --- | --- | --- | --- | --- |
| **ID** | **Plasmid (CTX-M-15)** | **TEM** | **TZP** | **CXM** | **CTX** | **CAZ** | **CZA** | **ATM** | **MEM** | **ERT** | **GEN** | **TOB** | **AMK** | **CIP** | **COL** | **SXT** |
| K012 | pK012_02^[[2]](#footnote-2)^ | <4 | 16 | >16 | >16 | 16 | 0,25 | >16 | 0,03 | 0,03 | 32 | 4 | <1 | 0,03 | 1 | >16 |
| K006 | pK012_02-like | <4 | 64 | >16 | >16 | 16 | 0,25 | >16 | 0,06 | 0,06 | 32 | 4 | <1 | 0,03 | 1 | >16 |
| K033 | pK012_02-like | 8 | >64 | >16 | >16 | 32 | 4 | >16 | 0,06 | 0,12 | 128 | 16 | <1 | 0,03 | 1 | >16 |
| K039 | pK012_02-like | <4 | 4 | >16 | >16 | 16 | 0,12 | 16 | 0,03 | 0,03 | 32 | 2 | <1 | 0,03 | 1 | >16 |
| K020 | pK020_1^[[3]](#footnote-3)^ | 8 | >64 | >16 | >16 | >32 | 0,5 | >16 | 0,03 | 0,06 | 128 | 16 | <1 | 0,06 | 1 | >16 |

1. TEM, temocillin; TZP, piperacillin/tazobactam; CXM, cefuroxime; CTX, cefotaxime; CAZ, ceftazidime; CZA, ceftazidime-avibactam; ATM, aztreonam; MEM, meropenem; ERT, ertapenem; GEN, gentamicin; TOB, tobramycin; AMK, amikacin; CIP, ciprofloxacin; COL, colistin; SXT, trimethoprim/sulfamethoxazole [↑](#footnote-ref-1)
2. Plasmid contains the following resistance encoding genes: *bla*_TEM-1B_; *aac*(3)*-*IId: *bla*_CTX-M-15_; *dfrA30*; *sul2* and *catA2* [↑](#footnote-ref-2)
3. Plasmid contains the following resistance encoding genes: *bla*_TEM-1B_; *aac*(3)-Iia; *bla*_CTX-M-15_; *bla*_SCO-1_ [↑](#footnote-ref-3)
