# Supplementary material for: Horizontal Plasmid Transfer among Klebsiella pneumoniae Isolates Is the Key Factor for Dissemination of Extended-Spectrum β-Lactamases among Children in Tanzania
Source: mSphere. 2020 Jul 15;5(4):e00428-20. doi: 10.1128/mSphere.00428-20 (PMC7364214; doi:10.1128/mSphere.00428-20)
Supplement: TABLE S4 [file mSphere.00428-20-st004.docx]

| **TABLE S4** Whole genome sequencing resultss and NCBI accession numbers for the *K. pneumoniae* strains | | | | | |
| --- | --- | --- | --- | --- | --- |
| **Strain** |  | **Illumina sequencing** | | | |
| **ID** | **BioSample** | **Instrument** | **# contigs** | **Coverage** | **Sequence Run** |
| K001 | SAMN10390440 | HiSeq | 139 | **70** | SRR8174951 |
| K002 | SAMN10390441 | MiSeq | 115 | 38 | SRR8174950 |
| K003 | SAMN10390442 | HiSeq | 161 | 116 | SRR8174953 |
| K004 | SAMN10390443 | HiSeq | 232 | 102 | SRR8174952 |
| K005 | SAMN10390444 | HiSeq | 151 | 121 | SRR8174955 |
| K006 | SAMN10390445 | HiSeq | 109 | 90 | SRR8174954 |
| K007 | SAMN10390446 | HiSeq | 89 | 97 | SRR8174957 |
| K008 | SAMN10390447 | HiSeq | 120 | 110 | SRR8174956 |
| K009 | SAMN10390448 | HiSeq | 254 | 83 | SRR8174949 |
| K010 | SAMN10390449 | HiSeq | 151 | 78 | SRR8174948 |
| K011 | SAMN10390450 | HiSeq | 141 | 56 | SRR8174961 |
| K012 | SAMN10390451 | HiSeq | 132 | 115 | SRR8174960 |
| K013 | SAMN10390452 | HiSeq | 161 | 126 | SRR8174959 |
| K015 | SAMN10390453 | HiSeq | 65 | 84 | SRR8174958 |
| K016 | SAMN10390454 | MiSeq | 131 | 42 | SRR8174965 |
| K017 | SAMN10390455 | HiSeq | 131 | 36 | SRR8174964 |
| K018 | SAMN10390456 | HiSeq | 137 | 98 | SRR8174963 |
| K020 | SAMN10390457 | HiSeq | 137 | 109 | SRR8174962 |
| K021 | SAMN10390458 | HiSeq | 146 | 118 | SRR8174967 |
| K022 | SAMN10390459 | HiSeq | 91 | 109 | SRR8174966 |
| K023 | SAMN10390460 | HiSeq | 140 | 125 | SRR8175020 |
| K024 | SAMN10390461 | HiSeq | 106 | 102 | SRR8175021 |
| K025 | SAMN10390462 | HiSeq | 110 | 96 | SRR8175018 |
| K026 | SAMN10390463 | HiSeq | 113 | 95 | SRR8175019 |
| K028 | SAMN10390464 | MiSeq | 155 | 41 | SRR8175024 |
| K029 | SAMN10390465 | MiSeq | 81 | 56 | SRR8175025 |
| K030 | SAMN10390466 | MiSeq | 142 | 42 | SRR8175022 |
| K031 | SAMN10390467 | MiSeq | 104 | 58 | SRR8175023 |
| K032 | SAMN10390468 | MiSeq | 130 | 44 | SRR8175016 |
| K033 | SAMN10390469 | MiSeq | 54 | 48 | SRR8175017 |
| K034 | SAMN10390470 | MiSeq | 62 | 37 | SRR8174999 |
| K035 | SAMN10390471 | MiSeq | 69 | 28 | SRR8174998 |
| K036 | SAMN10390472 | MiSeq | 179 | 35 | SRR8175001 |
| K037 | SAMN10390473 | MiSeq | 143 | 58 | SRR8175000 |
| K038 | SAMN10390474 | MiSeq | 81 | 35 | SRR8175003 |
| K039 | SAMN10390475 | MiSeq | 77 | 34 | SRR8175002 |
| K040 | SAMN10390476 | MiSeq | 74 | 37 | SRR8175005 |
| K041 | SAMN10390477 | MiSeq | 82 | 51 | SRR8175004 |
| K042 | SAMN10390478 | MiSeq | 84 | 46 | SRR8174997 |
| K043 | SAMN10390479 | MiSeq | 128 | 33 | SRR8174996 |
| K044 | SAMN10390480 | MiSeq | 86 | 49 | SRR8174900 |
| K045 | SAMN10390481 | MiSeq | 73 | 40 | SRR8174901 |
| K046 | SAMN10390482 | MiSeq | 142 | 34 | SRR8174902 |
| K047 | SAMN10390483 | MiSeq | 91 | 32 | SRR8174903 |
| K049 | SAMN10390484 | MiSeq | 291 | 70 | SRR8174904 |
| K050 | SAMN10390485 | MiSeq | 67 | 59 | SRR8174905 |
| K051 | SAMN10390486 | MiSeq | 160 | 38 | SRR8174906 |
| K052 | SAMN10390487 | MiSeq | 153 | 59 | SRR8174907 |
| K053 | SAMN10390488 | MiSeq | 150 | 55 | SRR8174898 |
| K054 | SAMN10390489 | MiSeq | 126 | 91 | SRR8174899 |
| K055 | SAMN10390490 | MiSeq | 136 | 61 | SRR8175015 |
| K056 | SAMN10390491 | MiSeq | 146 | 62 | SRR8175014 |
| K057 | SAMN10390492 | MiSeq | 157 | 45 | SRR8175013 |
| K058 | SAMN10390493 | MiSeq | 124 | 34 | SRR8175012 |
| K059 | SAMN10390494 | MiSeq | 238 | 58 | SRR8175011 |
| K060 | SAMN10390495 | MiSeq | 134 | 33 | SRR8175010 |
| K061 | SAMN10390496 | MiSeq | 175 | 31 | SRR8175009 |
| K062 | SAMN10390497 | MiSeq | 139 | 42 | SRR8175008 |
| K063 | SAMN10390498 | MiSeq | 95 | 32 | SRR8175007 |
| K064 | SAMN10390499 | MiSeq | 74 | 41 | SRR8175006 |
| K065 | SAMN10390500 | MiSeq | 168 | 40 | SRR8174944 |
| K066 | SAMN10390501 | MiSeq | 141 | 22 | SRR8174945 |
| K067 | SAMN10390502 | MiSeq | 144 | 53 | SRR8174942 |
| K068 | SAMN10390503 | MiSeq | 165 | 75 | SRR8174943 |
| K069 | SAMN10390504 | MiSeq | 107 | 34 | SRR8174940 |
| K070 | SAMN10390505 | MiSeq | 77 | 50 | SRR8174941 |
| K072 | SAMN10390506 | MiSeq | 139 | 47 | SRR8174938 |
| K073 | SAMN10390507 | MiSeq | 67 | 40 | SRR8174939 |
| K074 | SAMN10390508 | MiSeq | 154 | 31 | SRR8174946 |
| K075 | SAMN10390509 | MiSeq | 158 | 39 | SRR8174947 |
| K076 | SAMN10390510 | MiSeq | 118 | 40 | SRR8174923 |
| K077 | SAMN10390511 | MiSeq | 328 | 38 | SRR8174922 |
| K078 | SAMN10390512 | MiSeq | 316 | 29 | SRR8174925 |
| K079 | SAMN10390513 | MiSeq | 180 | 40 | SRR8174924 |
| K080 | SAMN10390514 | MiSeq | 138 | 37 | SRR8174919 |
| K081 | SAMN10390515 | MiSeq | 119 | 35 | SRR8174918 |
| K082 | SAMN10390516 | MiSeq | 112 | 39 | SRR8174921 |
| K083 | SAMN10390517 | MiSeq | 149 | 40 | SRR8174920 |
| K084 | SAMN10390518 | MiSeq | 134 | 28 | SRR8174927 |
| K085 | SAMN10390519 | MiSeq | 149 | 40 | SRR8174926 |
| K086 | SAMN10390520 | MiSeq | 134 | 34 | SRR8174982 |
| K087 | SAMN10390521 | MiSeq | 130 | 42 | SRR8174983 |
| K088 | SAMN10390522 | MiSeq | 196 | 26 | SRR8174984 |
| K089 | SAMN10390523 | MiSeq | 151 | 29 | SRR8174985 |
| K090 | SAMN10390524 | MiSeq | 155 | 46 | SRR8174978 |
| K091 | SAMN10390525 | MiSeq | 125 | 44 | SRR8174979 |
| K092 | SAMN10390526 | MiSeq | 141 | 31 | SRR8174980 |
| K093 | SAMN10390527 | MiSeq | 149 | 30 | SRR8174981 |
| K095 | SAMN10390528 | MiSeq | 59 | 37 | SRR8174986 |
| K096 | SAMN10390529 | MiSeq | 85 | 27 | SRR8174987 |
| K097 | SAMN10390530 | MiSeq | 197 | 23 | SRR8174973 |
| K098 | SAMN10390531 | MiSeq | 136 | 33 | SRR8174972 |
| K099 | SAMN10390532 | MiSeq | 72 | 22 | SRR8174971 |
| K100 | SAMN10390533 | MiSeq | 84 | 35 | SRR8174970 |
| K101 | SAMN10390534 | MiSeq | 186 | 40 | SRR8174977 |
| K102 | SAMN10390535 | MiSeq | 287 | 22 | SRR8174976 |
| K103 | SAMN10390536 | MiSeq | 109 | 22 | SRR8174975 |
| K104 | SAMN10390537 | MiSeq | 160 | 18 | SRR8174974 |
| K105 | SAMN10390538 | MiSeq | 76 | 25 | SRR8174969 |
| K106 | SAMN10390539 | MiSeq | 180 | 27 | SRR8174968 |
| K107 | SAMN10390540 | MiSeq | 114 | 20 | SRR8174910 |
| K108 | SAMN10390541 | MiSeq | 130 | 32 | SRR8174911 |
| K109 | SAMN10390542 | MiSeq | 198 | 29 | SRR8174908 |
| K110 | SAMN10390543 | MiSeq | 230 | 22 | SRR8174909 |
| K111 | SAMN10390544 | MiSeq | 139 | 35 | SRR8174914 |
| K112 | SAMN10390545 | MiSeq | 145 | 22 | SRR8174915 |
| K113 | SAMN10390546 | MiSeq | 154 | 29 | SRR8174912 |
| K114 | SAMN10390547 | MiSeq | 106 | 65 | SRR8174913 |
| K115 | SAMN10390548 | MiSeq | 116 | 41 | SRR8174916 |
| K116 | SAMN10390549 | MiSeq | 78 | 31 | SRR8174917 |
| K117 | SAMN10390550 | MiSeq | 157 | 50 | SRR8174929 |
| K118 | SAMN10390551 | MiSeq | 222 | 37 | SRR8174928 |
| K119 | SAMN10390552 | MiSeq | 138 | 44 | SRR8174931 |
| K120 | SAMN10390553 | MiSeq | 70 | 37 | SRR8174930 |
| K121 | SAMN10390554 | MiSeq | 94 | 41 | SRR8174933 |
| K122 | SAMN10390555 | MiSeq | 116 | 51 | SRR8174932 |
| K123 | SAMN10390556 | MiSeq | 156 | 44 | SRR8174935 |
| K124 | SAMN10390557 | MiSeq | 107 | 38 | SRR8174934 |
| K125 | SAMN10390558 | MiSeq | 150 | 31 | SRR8174937 |
| K126 | SAMN10390559 | MiSeq | 72 | 88 | SRR8174936 |
| K127 | SAMN10390560 | MiSeq | 134 | 37 | SRR8174988 |
| K128 | SAMN10390561 | MiSeq | 93 | 46 | SRR8174989 |
| K129 | SAMN10390562 | MiSeq | 95 | 50 | SRR8174990 |
| K130 | SAMN10390563 | MiSeq | 62 | 37 | SRR8174991 |
| K131 | SAMN10390564 | MiSeq | 91 | 31 | SRR8174992 |
| K132 | SAMN10390565 | MiSeq | 128 | 34 | SRR8174993 |
| K133 | SAMN10390566 | MiSeq | 146 | 31 | SRR8174994 |
| K134 | SAMN10390567 | MiSeq | 155 | 23 | SRR8174995 |
